# Engineering exosomes for targeted neurodegenerative therapy: innovations in biogenesis, drug loading, and clinical translation

**DOI:** 10.7150/thno.117143

**Published:** 2026-01-01

**Authors:** Qinqin Huang, Shile Wang, Zeming Liu, Lang Rao, Ke Cheng, Xiaobo Mao

**Affiliations:** 1 School of Electronic and Electrical Engineering, Wuhan Textile University, Wuhan 430200, China.; 2 Department of Plastic Surgery, Tongji Hospital, Tongji Medical College, Huazhong University of Science and Technology, Wuhan 430030, China.; 3 Institute of Chemical Biology, Shenzhen Bay Laboratory, Shenzhen 518132, China.; 4 Department of Biomedical Engineering, Columbia University, New York 10027, USA.; 5 Neuroregeneration and Stem Cell Programs, Institute for Cell Engineering, Johns Hopkins University School of Medicine, Baltimore, MD 21205, USA.; 6 Department of Neurology, Johns Hopkins University School of Medicine, Baltimore, MD 21205, USA.; 7 Adrienne Helis Malvin Medical Research Foundation, New Orleans, LA 70130-2685, USA.; 8 Institute for NanoBioTechnology, Johns Hopkins University, Baltimore, MD 21205, USA.; 9 Department of Materials Science and Engineering, Johns Hopkins University, Baltimore, MD 21205, USA.

**Keywords:** neurodegenerative diseases (NDDs), exosomes, blood-brain barrier (BBB), drug delivery, targeted therapy, clinical trials

## Abstract

Neurodegenerative diseases (NDDs), including Alzheimer's disease (AD), Parkinson's disease (PD), amyotrophic lateral sclerosis (ALS), Huntington's disease (HD) and multiple sclerosis (MS), are characterized by progressive neuronal dysfunction and limited therapeutic options, largely due to the restrictive nature of the blood-brain barrier (BBB). Exosomes, naturally occurring extracellular vesicles (EVs), have gained attention as innovative drug delivery vehicles owing to their intrinsic ability to cross the BBB, minimal immunogenicity, high biocompatibility, and capability to carry diverse therapeutic cargos such as proteins, nucleic acids, and small molecules. Furthermore, exosomes can be bioengineered to enhance drug-loading efficiency and targeting specificity, positioning them as a versatile and effective platform for treating NDDs. In this review, we summarize recent advances in exosome biogenesis, secretion, and engineering, with an emphasis on innovative strategies for exosome isolation, drug loading, and surface modification. We further explore their roles in modulating neuroinflammation, promoting neural regeneration, and enabling precise therapeutic delivery. Critical challenges associated with large-scale production, quality control, and regulatory compliance under Good Manufacturing Practices (GMP) are also discussed. Collectively, these developments underscore the transformative potential of engineered exosomes in advancing precision therapies for neurodegenerative disorders and offer strategic insights into their clinical translation.

## Introduction

With advancements in medical technology and improvements in living standards, population aging has become an irreversible global trend, and neurodegenerative diseases (NDDs) have emerged as a significant medical challenging in modern society. According to recent research published in *The Lancet Neurology*, by 2021, neurological disorders affected more than one-third of the global population and have become the leading cause of illness and disability 1. NDDs encompass a range of disorders characterized by progressive loss of neuronal structure and function in the central or peripheral nervous system, including Alzheimer's disease (AD) 2, Parkinson's disease (PD) 3, amyotrophic lateral sclerosis (ALS) 4,5, Huntington's disease (HD) 6, and multiple sclerosis (MS) 7. These conditions are often accompanied by cognitive impairment or motor dysfunction, and the damage is frequently irreversible due to the limited regenerative capacity of the nervous system 8,9. However, the underlying molecular and cellular mechanisms remain incompletely understood. Pathological hallmarks, including abnormal protein aggregation, mitochondrial dysfunction, neuroinflammation, and oxidative stress, provide insights into disease etiology and are central to the development of targeted therapeutic strategies 8.

However, most potential therapeutic drugs face difficulties in crossing the blood-brain barrier (BBB), and even when they do manage to cross, they often fail to reach therapeutic concentrations in the brain 10. Clinically, this has contributed to the lack of effective treatments for NDDs, making the development of novel delivery strategies essential. The advancement of nanotechnology has led to the development of various drug delivery systems, such as liposomes 11, solid lipid nanoparticles 12, and polymer nanoparticles 13, designed to facilitate intracerebral drug delivery. However, these approaches still face considerable limitations, including difficulties in large-scale production, rapid clearance by the body's mononuclear phagocyte system, and potential toxic side effects 14,15. This highlights the need for alternative delivery vehicles that can overcome these challenges. In this regard, natural nanovesicles, particularly exosomes, have emerged as highly promising drug carriers, providing several advantages over traditional nanoparticle systems (Table 1).

Exosomes, which range from ~40-160 nm in diameter, are naturally occurring vesicles derived from the endosomal system. These vesicles begin as early-sorting endosome formed by the invagination of the cytoplasmic membrane, which further undergo outgrowths to form multivesicular bodies (MVBs) 24-26. MVBs then fuse with the cytoplasmic membrane to release exosomes into the extracellular space, making this a critical step in intercellular communication. The distinct biogenesis process and cargo-delivery capabilities of exosomes have increasingly positioned them as promising drug carriers in various biomedical applications.

As drug carriers, exosomes offer several advantages over conventional delivery systems. Their low immunogenicity, toxicity, high biocompatibility, and stability make them ideal for treating chronic conditions like NDDs, where long-term, targeted drug delivery is crucial. Moreover, exosomes can be produced on a large scale, addressing a key challenge in traditional drug delivery. Their natural ability to target specific tissues, including the capacity to cross biological barriers such as the BBB, makes them especially promising for treating NDDs, where efficient drug delivery to the brain remains a significant challenge 26-29. Additionally, exosomes can be engineered to carry a variety of therapeutic payloads—ranging from proteins and nucleic acids to gene-editing tools, adeno-associated viruses, and small molecule drugs—making them versatile platforms for diverse biomedical applications 26,30.

The unique structural properties of exosomes, along with their efficient secretion mechanisms, enable them to encapsulate a broad range of bioactive molecules, which can be delivered precisely to targeted sites within the nervous system. Exosomes are involved in several critical processes that regulate nervous system function. They can promote neuronal growth, differentiation, and remodeling, as well as inhibit neuroinflammation and undesired immune responses. Furthermore, exosomes contribute to the enhancement of the vascular network in the nervous system and can modulate the integrity and permeability of the BBB—a key consideration in the treatment of NDDs, where crossing the BBB is often a major obstacle 31-34. This ability to deliver bioactive molecules—either naturally present or therapeutically loaded—enables exosomes to regulate gene expression and related signaling pathways, thereby influencing neuronal structure and function in ways that support the repair or regeneration of neural tissue 31.

**Figure 1:**
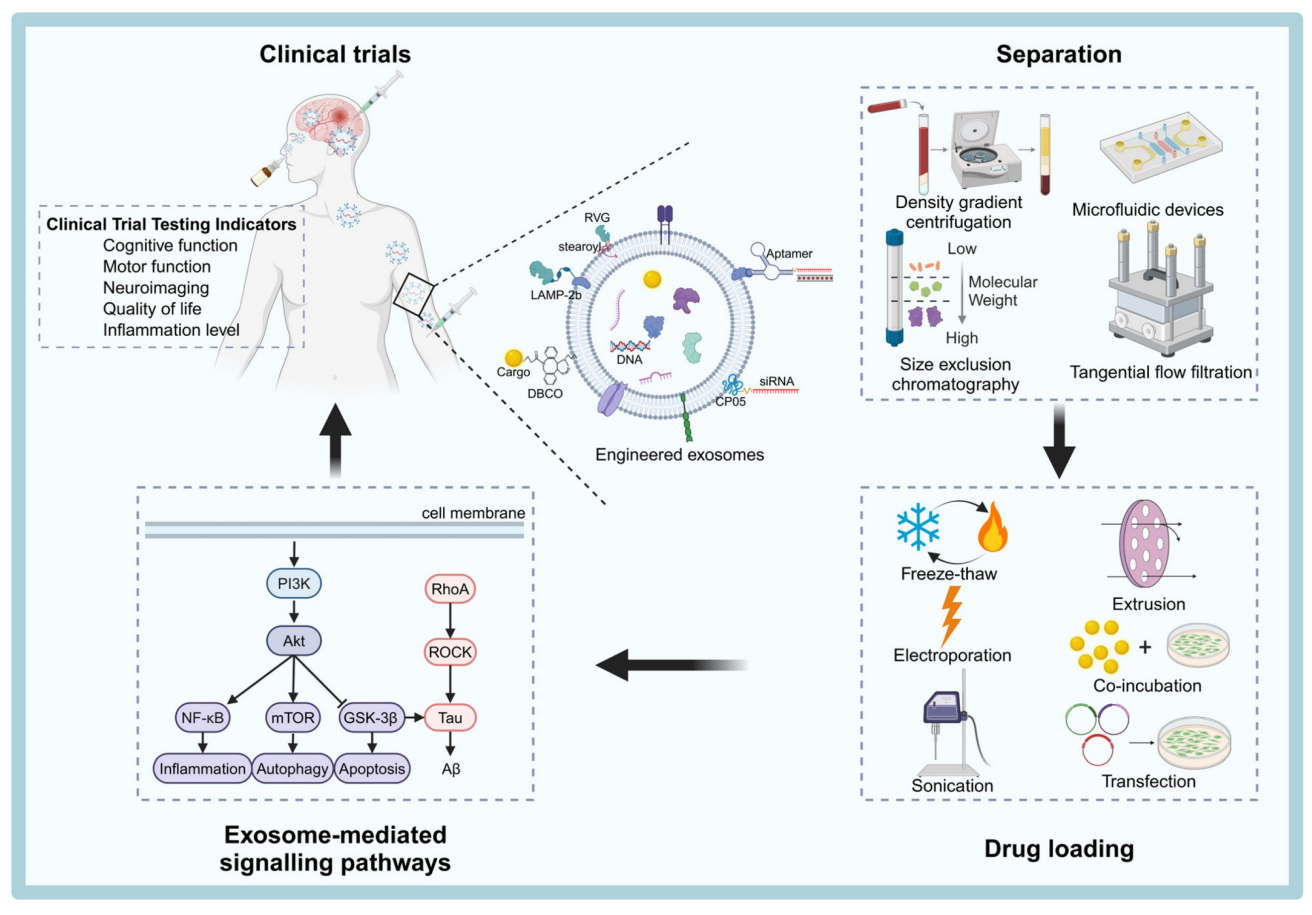
**Schematic overview of the process of engineering exosomes for therapeutic applications of NDDs.** Natural exosomes are first isolated from body fluids, and then they are engineered to carry specific cargoes or express ligands on their surfaces that can be clinically therapeutic by targeting pathways. The figure was created with BioRender.

This review aims to provide a comprehensive summary of the latest advances in exosome biogenesis, secretion, isolation techniques, surface modification strategies, and drug-loading techniques. Additionally, we will explore the signaling pathways targeted by exosomes in the treatment of NDDs and provide an overview of the status of exosome-based therapies. Finally, we will discuss the challenges that remain in translating exosome technologies from the laboratory to clinical applications and propose potential solutions to overcome these hurdles (Figure 1).

## Exosome Biogenesis, Sources, and Therapeutic Potential in NDDs

### Recent Advances in Exosome Biogenesis and Secretion Mechanisms

Exosomes are small vesicles secreted by cells that play crucial roles in intercellular signaling. They are involved in various physiological and pathological processes, such as immune response modulation, tumor progression, and the pathogenesis of NDDs. In recent years, exosomes have gained significant attention due to their potential in disease prevention, diagnosis, treatment, and prognostic assessment. A deeper understanding of the biogenesis and secretion mechanisms of exosomes, as well as the regulation of their key molecular targets, not only provides new therapeutic strategies but also lays the foundation for optimizing exosomes for clinical applications, especially in the context of NDDs. In the following, we provide an updated overview of recent advancements in the biogenesis and secretion mechanisms of exosomes, highlighting key molecular players and novel discoveries that contribute to the regulation of exosome release.

The Rab GTPase family is a critical regulator of exosome secretion. Rab GTPases influence exosome release by modulating the transport of MVBs or their docking with the plasma membrane of target cells 35. Among the various Rab GTPases, Rab27a has been extensively studied and shown to be crucial in the fusion of MVBs with the plasma membrane, facilitating the release of exosomes 36. Recently, Song *et al.* 37 revealed that knockdown of Kidney Brain Expressed Protein (KIBRA) in neuronal and podocyte cell lines led to reduced exosome secretion, despite an increase in the size and number of intracellular MVBs. Additionally, Rab27a expression was significantly diminished in KIBRA knockout mice. These findings suggest that KIBRA may facilitate exosome membrane trafficking by binding to Rab27a and regulating its degradation, thus playing a key role in exosome secretion. This mechanism could play an important role in the pathogenesis and progression of NDDs by affecting the release of neurodegeneration-associated molecules.

While the role of Rab GTPases in exosome secretion is well-established, the molecular mechanisms governing MVBs transport to the plasma membrane for exosome secretion, as opposed to lysosomal degradation, remain unclear. This gap in knowledge has sparked interest in other protein complexes involved in this process.

The Exocyst complex, a conserved octameric protein complex localized at the dynamic regions of the plasma membrane, has been implicated in exosome transport. It facilitates membrane fusion through the activation of soluble N-ethylmaleimide-sensitive factor attachment proteins 38. Liu *et al.* 39 discovered that the conversion of phosphatidylinositol-3-phosphate to phosphatidylinositol-4-phosphate on MVBs is necessary for the recruitment of the Exocyst complex, which in turn promotes the outward transport and exocytosis of MVBs. Moreover, Lee *et al.* 40 provided further evidence that the Exocyst complex plays a critical chaperone role during SNARE complex assembly and membrane fusion, thereby regulating cytokinesis at multiple stages. These findings suggest that the Exocyst complex acts as a chaperone to facilitate the fusion of MVBs with the plasma membrane, an essential step in exosome release.

The process of exosome biogenesis is intricately linked to endocytosis, as exosomal proteins are often delivered to early endosomes via endocytic pathways. These proteins are then incorporated into nascent intraluminal vesicles (ILVs) within MVBs, which later fuse with the plasma membrane to release exosomes 24,41. However, recent studies have shown that endocytosis can play a dual role in exosome secretion. Gao *et al.* 42 found that endocytosis strongly inhibited the secretion of exosomal marker proteins such as CD81, CD9, and CD63 and triggered their degradation in the presence of CD81 and CD9. The study also revealed that syntenin proteins promote the vesicular secretion of CD63 by blocking its endocytosis, shedding light on the complex relationship between endocytosis and the secretion of exosomal marker proteins. This finding introduces a new layer of complexity in our understanding of how exosomal marker proteins are regulated.

In addition, recent studies have highlighted the close relationship between exosome formation and the autophagic process, though the exact mechanisms remain incompletely understood 43. Yanagawa *et al.* 44 conducted RNA interference screening of autophagy-related factors and found that Rubicon, a cysteine-rich domain-containing protein, positively regulates exosome biogenesis in an autophagy-independent manner. Further investigation showed that Rubicon regulates the activation of the endosomal sorting complexes required for transport through the Rubicon-WIPI axis, thereby precisely controlling exosome production and secretion. This discovery provides new insights into the molecular mechanisms of exosome biosynthesis and could offer novel strategies for utilizing exosomes in disease therapy.

In summary, the mechanisms underlying exosome production and secretion involve multiple key molecular and cellular processes, including Rab GTPases, the Exocyst complex, and the interplay between endocytosis and autophagy. These studies not only enhance our understanding of exosomes in intercellular communication but also open new avenues for their application in disease diagnosis and treatment. By fine-tuning the regulation of exosome biogenesis, particularly the secretion of membrane proteins, we can pave the way for breakthrough therapies in various diseases, including cancer and NDDs. The continued exploration of exosome biology will likely lead to novel strategies for using these vesicles in precision medicine and targeted drug delivery.

### Exosome Sources and Their Therapeutic Specificity in NDDs

Exosomes, as nanosized EVs secreted by nearly all cell types, serve as pivotal mediators of intercellular communication by transferring diverse biological cargoes including proteins, RNAs, and lipids. In the context of NDDs, exosomes modulate key pathological processes such as neuroinflammation, neuronal survival, synaptic plasticity, and tissue repair. Consequently, therapeutic exosomes have garnered significant interest as innovative drug delivery systems and immunomodulatory agents.

Crucially, the therapeutic potential of exosomes is inherently linked to their cellular origin. Different cell types impart distinct molecular cargos, surface markers, and functional properties to their secreted exosomes. These differences not only determine the exosomes' ability to target specific cell types or tissues, but also influence their immunogenicity, safety profile, and regenerative potential. Understanding how the source of exosomes shapes their characteristics is essential for tailoring effective treatments in NDDs (Table 2).

#### Mesenchymal Stem Cells (MSCs)

**Figure 2:**
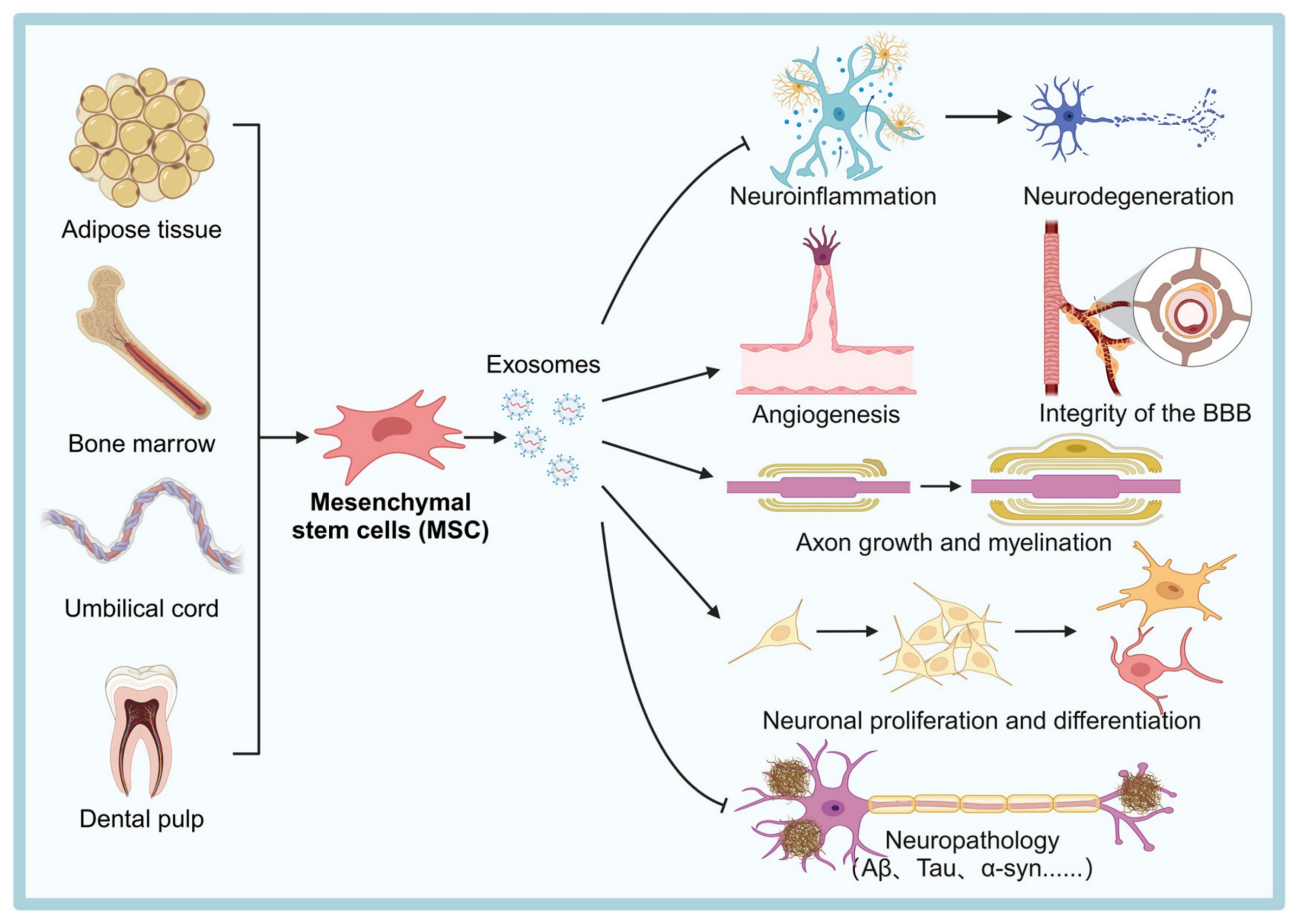
** Sources of MSCs and the potential mechanisms of MSC-Exos therapy for the treatment of NDDs.** MSC-Exos help reduce neuroinflammation, promote angiogenesis, enhance axon growth and myelination, support neuronal proliferation and differentiation, and maintain the integrity of the BBB, ultimately alleviating neurodegeneration and related neuropathology. The figure was created with BioRender.

MSCs are one of the most researched and clinically used sources of therapeutic exosomes. They can be isolated from various tissues such as umbilical cord, dental pulp, adipose tissue, and bone marrow, and are widely used in the treatment of NDDs 54 (Figure 2). MSCs-derived exosomes (MSC-Exos) are enriched with active substances such as trophic factors, anti-inflammatory cytokines (e.g., interleukin (IL)-10, transforming growth factor (TGF)-β), and regulatory miRNAs (e.g., miR-21, miR-146a), which can exert neuroprotective, immunomodulatory, and pro-angiogenic effects 55. For instance, injection of bone marrow MSC exosomes (BMSC-Exos) into the lateral ventricle of mice significantly modulated hippocampal neuroinflammation, increased the expression of brain-derived neurotrophic factor (BDNF), improved synaptic plasticity and reduced the expression of neuroinflammatory plaques and abnormal expression of phosphorylated tau (P-Tau) 45.

Furthermore, growth differentiation factor-15 secreted by human umbilical cord blood MSCs (hUCB-MSCs) promotes hippocampal neuronal proliferation, differentiation and synaptic activity 56. In a mouse model of experimental autoimmune encephalomyelitis, γ-interferon (IFNγ)-stimulated MSC-Exos improved motor function, reduced neuroinflammation, and attenuated demyelination injury 57. Additionally, miR-146a-5p-modified hUCB-MSCs exosomes reduced the toxic effects of neurotoxic astrocytes and promoted the recovery of neurological function in spinal cord injured rats 47. These studies highlight the potential of MSC-Exos in the treatment of neuroinflammation and nerve injury. Regarding angiogenesis, MSC-Exos promote angiogenesis in human brain microvascular ECs 58. They enhance ECs proliferation and migration by delivering microRNAs, such as miR-21-5p, which promotes angiogenesis and ameliorates cerebral ischemic injury in an ischemic stroke mouse model 59. These effects may also help repair BBB damage and restore its function.

In terms of targeting ability, MSC-Exos express surface proteins such as integrins and tetraspanins, which help them to cross the BBB and preferentially homing to sites of injury or inflammation. For example, Perets *et al.* 60 using tomography with gold nanoparticle-labeled MSC-Exos, found that these exosomes specifically targeted and accumulated in the brain of a mouse model with associated pathological changes, suggesting a significant ability to migrate and home to neurons. MSC-Exos possess low immunogenicity, high biocompatibility, neuroprotective properties, and the ability to be surface-engineered for enhanced targeting. These characteristics collectively provide a strong molecular and biological foundation for their potential clinical applications in NDDs and other diseases. Although MSC-Exos show great therapeutic promise, their clinical translation still faces challenges, mainly including: relatively high production costs, difficulties in achieving consistent purity from batch to batch, and the lack of standardized dosing regimens.

#### Neural stem cells (NSCs) and neurons

NSCs possess self-renewal capability and can differentiate into neuronal and glial lineages. NSCs-derived exosomes (NSC-Exos) inherit abundant neuroprotective and neuroregenerative non-coding RNAs, proteins, lipids, and other active constituents from the donor cells, and exhibit excellent neuroprotective and neuroregenerative potentials, as well as immunomodulatory capabilities 61. For example, NSC-Exos have been shown to enhance mitochondrial function, activate sirtuin 1, increase synaptic activity, reduce neuroinflammation, and ameliorate disease progression in a mouse model of AD 49. Owing to its donor cell properties, NSC-Exos have a high degree of targeting specificity to neuronal cells and is capable of precise interactions with neurons and glial cells. Despite the significant advantages of low immunogenicity and high specificity of NSC-Exos in neurotargeting therapy, their clinical translation still faces major obstacles, such as limited donor cells and difficulties in scale-up production.

#### Induced Pluripotent Stem Cells (iPSCs)

iPSCs possess unlimited self-renewal capacity and the ability to differentiate into nearly all cell types. iPSCs-derived exosomes (iPSC-Exos) are rich in growth factors, developmental regulators, and miRNAs associated with pluripotency and tissue regeneration, enabling them to alleviate vascular aging, reduce neuroinflammation, and promote nerve regeneration. For example, Niu *et al.* 62 found that iPSC-EVs could attenuate microglia senescence and promote the shift of microglia polarization from a pro-inflammatory phenotype to an anti-inflammatory phenotype, which protects neurons from death and improves the prognosis of ischemic stroke in the elderly. In terms of targeting, undifferentiated iPSC-Exos exhibit broad tropism, relying mainly on their surface-carried adhesion molecules and integrins, to bind to receptors prevalent at the site of injury. iPSCs can be expanded indefinitely, which overcomes the problems of the limited source of progenitor cells (e.g., MSCs, neurons) and the large inter-batch variability, and provides the possibility of large-scale, standardized production of clinical-grade exosomes. However, their application to clinical practice is constrained by manufacturing complexity, high production costs, and safety issues related to their pluripotent origins. In addition, stringent quality control measures are required to ensure consistency and safety.

#### Endothelial cells (EC)

ECs play a critical role in vascular regeneration and tissue repair through their proliferative and migratory capabilities, contributing to new blood vessel formation and secretion of growth factors and cytokines (e.g., vascular endothelial growth factor, fibroblast growth factor) 63. ECs-derived exosomes (EC-Exos) contain bioactive substances such as growth factors, cytokines, and pro-angiogenic microRNAs, which mimic some parental cell functions and exert protective effects on neuronal cells by promoting cell growth, migration, and inhibiting apoptosis. For example, a study by Huang *et al.* 52 demonstrated that EC-Exos can effectively promote and maintain the repair-related phenotype of Schwann cells, thereby significantly enhancing axonal regeneration, myelin formation, and functional recovery of damaged nerves. Although the easy cultivation of exosomes and their ability to promote vascular repair are their obvious advantages, their limited neural targeting ability and potential immunogenicity may limit their application in direct neurotherapy.

#### Exosomes from other sources and body fluids

Beyond cell culture-derived exosomes, exosomes isolated from body fluids such as blood, urine, bile, saliva, cerebrospinal fluid (CSF), amniotic fluid, and breast milk offer unique insights and therapeutic opportunities 26. Studies have shown that exosomes from different body fluids vary in their ability to cross the BBB and their therapeutic potential 64. For instance, exosomes from saliva and urine have a limited ability to cross the BBB and are generally not used for targeting the brain in drug delivery. However, CSF-derived exosomes typically exhibit higher neurospecificity and can directly reflect the pathological states within the brain, making them more suitable as carriers for delivering therapeutic cargoes to brain tissues in conditions like NDDs, brain tumors, or spinal cord injuries.

Despite their potential, direct isolation of exosomes from CSF is challenging due to the complex and painful procedures involved, which limits their widespread use. However, CSF-derived exosomes can cross the BBB and diffuse through the bloodstream into other body fluids, such as blood and urine. As a result, brain-derived exosomes can be purified from body fluids by targeting specific surface markers. For example, brain-derived exosomes have been extracted using immunoprecipitation, employing the "ExoQuick" commercial exosome precipitation kit combined with anti-human CD171 antibodies 65. Since brain-derived exosomes are present in low quantities in other body fluids and their isolation and purification are complex and time-consuming, researchers have explored large-scale methods for isolating brain-derived exosomes by culturing neural cells. Exosomes secreted by cultured neural cells, such as neurons or NSCs, can serve as effective carriers for treating neurological diseases. For instance, Madhu *et al.* 50 successfully obtained exosomes derived from human iPSC (hiPSC)-derived NSCs (hiPSC-NSCs). Their experiments showed that hiPSC-NSC-EVs induced changes in the transcriptome of microglia and reactive astrocytes, which suppressed neuroinflammatory signaling in an AD model and reduced the accumulation of amyloid-β plaques and P-Tau.

In conclusion, the therapeutic efficacy and safety of exosomes in NDDs are intrinsically linked to their cellular origin, which governs their molecular cargo, targeting specificity, and immunogenic profile. Currently, MSC-Exos, NSC- Exos, and CSF-derived exosomes emerge as leading candidates for clinical translation in NDDs therapy. Future efforts should focus on optimizing large-scale production, enhancing targeting specificity via surface engineering, improving cargo loading techniques, and establishing robust quality control protocols. Such advances will pave the way for realizing the full therapeutic potential of exosomes, ultimately transforming the landscape of neurodegenerative disease treatment.

## Advances in Exosome Isolation and Drug-Carrying Technologies

The low yield and purity of exosomes remain significant barriers to their widespread clinical application. The clinical production of exosomes as delivery vehicles requires removing a large number of impurities, including cellular debris, proteins, free nucleic acids, and non-exosomal EVs (e.g., apoptotic vesicles and microvesicles) 66. Moreover, the size and physicochemical properties of exosomes often overlap with those of lipoproteins and other extracellular particles, further complicating the isolation and purification process.

**Figure 3:**
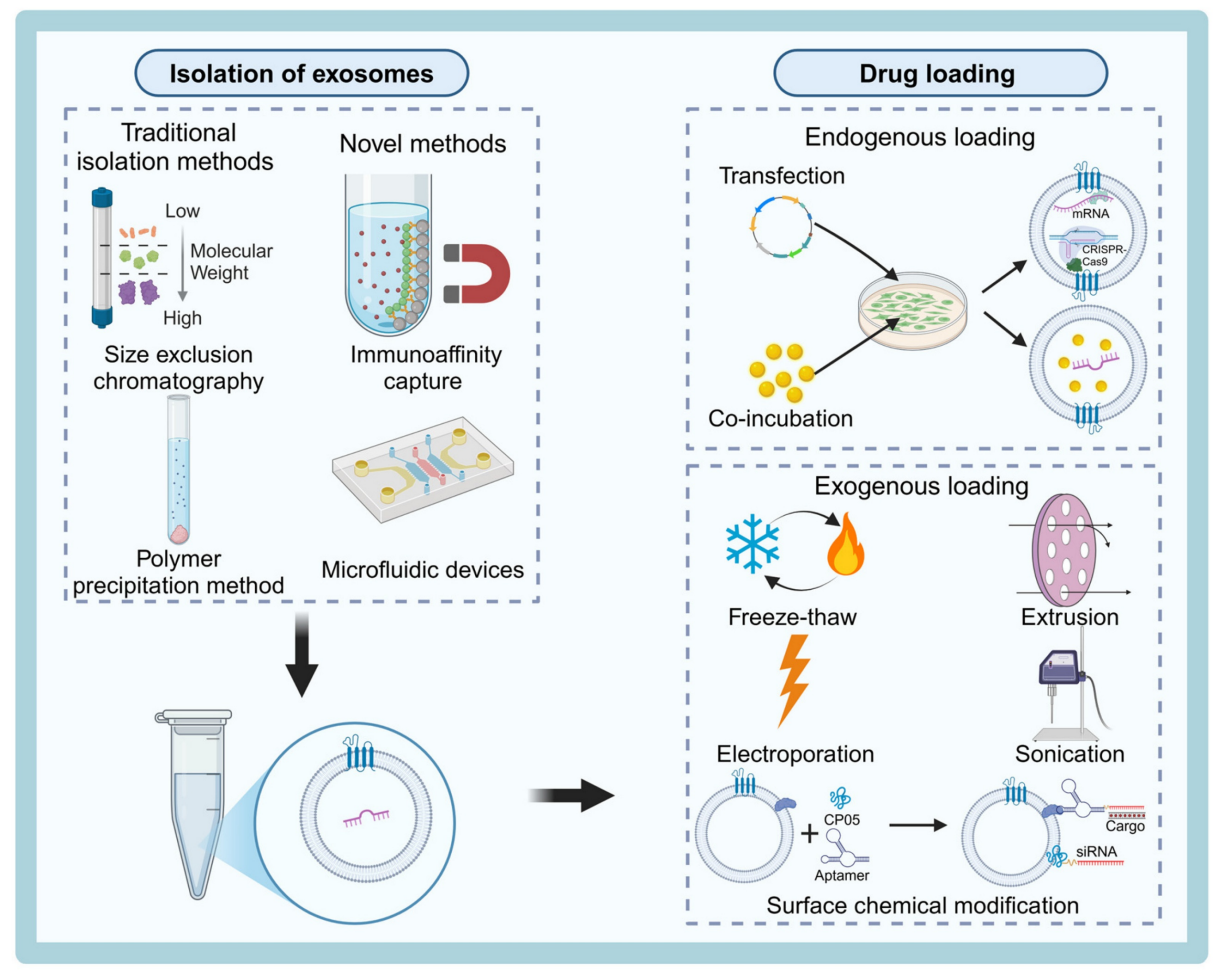
** Schematic diagram of the process for isolation and drug loading of engineered exosomes.** Exosomes can be isolated using traditional methods such as size exclusion chromatography and polymer precipitation, or novel techniques like immunoaffinity capture and microfluidic devices. Drug loading approaches include endogenous methods (e.g., transfection and co-incubation) and exogenous methods (e.g., freeze-thaw, extrusion, electroporation, and sonication), along with surface chemical modifications to incorporate therapeutic cargoes such as siRNA or CRISPR components. The figure was created with BioRender.

Currently, several commonly used methods for exosome isolation are available at the laboratory scale, including differential ultracentrifugation, density gradient centrifugation, ultrafiltration, polymer-based immunoprecipitation, size exclusion chromatography (SEC), immunoaffinity capture technology, and microfluidic technology (Table 3). Despite these available techniques, there is still no consensus in the field regarding the best approach for large-scale production of exosomes. Significant technical bottlenecks remain, particularly in achieving high yields and purity. Therefore, efficient isolation and purification of exosomes remains a critical area of research. Most conventional isolation methods rely on the physical properties of exosomes, such as size, density, surface charge, and immunoaffinity 67 (Figure 3).

### Traditional Isolation Methods vs. Novel Methods

#### Exosome isolation based on ultracentrifugation method

Ultracentrifugation is a classical physical isolation technique that separates exosomes from other components based on their different settling velocities under centrifugal force. This technique can be broadly divided into differential ultracentrifugation and density gradient ultracentrifugation.

Differential ultracentrifugation is considered the “gold standard” for exosome extraction. In this process, exosomes, cellular debris, proteins, and other impurities are separated by centrifugal force due to differences in their size and density 81. Teixeira-Marques *et al.* 82 optimized the ultracentrifugation process to reduce processing time and increase the yield of small EVs. They found that extending the centrifugation time at 200,000 × g could enhance EVs recovery. Studies have shown that factors such as centrifugation time, centrifugal force, rotor type, and other operational parameters influence both the yield and purity of the isolated exosomes. The ultrahigh-speed centrifugation involved can also damage the exosome structure, potentially affecting their function and biological activity, rendering it impractical for large-scale production.

Density gradient ultracentrifugation enhances the separation efficiency by introducing a density gradient (typically using sucrose) to isolate exosomes from particles with similar size but different densities. Exosomes typically accumulate in the density range of 1.13-1.19 g/mL, allowing them to be isolated from other particles 83. A notable advancement in this method is the one-step sucrose-buffered ultracentrifugation technique developed by Gupta *et al.* 70, which improves both the purity and yield of exosomes. However, the use of sucrose as a gradient medium has limitations, including its toxicity and suboptimal separation efficiency.

Iodixanol is a highly hydrophilic, nonionic substance with low viscosity and cytotoxicity, making it less toxic to cells compared to sucrose. Additionally, iodixanol provides better separation due to its stability and finer gradient resolution. Li *et al.* 84 introduced a stabilized platform for buffered density gradient ultracentrifugation using a 60% iodixanol pad to concentrate exosomes and effectively remove protein contaminants and non-exosomal nanoparticles. Moreover, D'Acunzo *et al.* 85 successfully isolated different EV populations from mouse brain tissue using iodixanol-based high-resolution density gradients, demonstrating better resolution and efficiency in separating microvesicles, exosomes, and mitochondrial vesicles.

Despite its advantages, isodensity gradient ultracentrifugation relies solely on the density differences between the solutes in the sample. It cannot effectively separate substances with similar densities to exosomes, such as microbubbles. Furthermore, the repetitive centrifugation and high centrifugal forces involved may cause mechanical damage to exosomes, which could impact subsequent functional studies and applications in drug development.

#### Size-based isolation method

Size-based isolation techniques, such as ultrafiltration and SEC, leverage differences in particle size to isolate exosomes from smaller or larger contaminants. These methods provide high efficiency and lower operational complexity compared to ultracentrifugation.

Ultrafiltration is a pressurized membrane separation technique that isolates exosomes from free nucleic acids, lipoproteins, and other components based on their size. The principle of tangential flow filtration (TFF) involves the tangential flow of the sample across the membrane, which reduces damage to flexible structures like exosomes, minimizes the risk of membrane clogging, and improves filtration efficiency 86. For example, Hou *et al.* 87 developed an electric field-assisted TFF device (E-TFF), which combines size-based filtration and electrophoretic migration separation techniques to achieve high throughput and high purity separation of small EVs. This method enhances separation efficiency and improves exosome integrity, making it a promising technique for both laboratory and commercial applications 88.

To further enhance the ultrafiltration process, Chen *et al.* 89 developed a fast and ultrafast separation system (EXODUS), which purifies exosomes from various biofluids automatically and without labels. By introducing negative-pressure oscillations and dual-coupled harmonic oscillations in a dual-membrane filter configuration, EXODUS suppresses fouling and particle aggregation, improving separation speed, yield, and purity while extending membrane lifespan. Moreover, Kim *et al.* 90 developed a bi-directional flow filtration system (BFF) based on direct flow filtration, integrating a backwash function. By employing 200 nm and 50 nm pore size membranes, BFF selectively isolates exosomes and prevents clogging through periodic backwashing. The BFF technique demonstrated 26-fold and 19-fold improvements in purity and recovery compared to ultracentrifugation and DFF, respectively, highlighting its potential for large-scale separations.

In comparison to ultracentrifugation, ultrafiltration offers several advantages, such as easier operation, higher enrichment efficiency, and lower cost 91. Although ultrafiltration has shown significant success in laboratory and small-scale applications, there are still challenges to overcome in scaling up for commercial use. Key issues include selecting membranes suited for large-scale filtration, developing more efficient automated systems for membrane replacement and operation, and real-time monitoring and adjustment of operating parameters. Additionally, maintaining a consistent shear force while ensuring effective filtration efficiency during industrial-scale production remains a challenge.

SEC is a technique that utilizes particle size differences to separate substances through porous polymer gel packing. Unlike ultracentrifugation, which subjects exosomes to high g-forces that can compromise membrane integrity and degrade RNA content, SEC employs gentle gravity-driven flow, thus better preserving exosomal morphology and ensuring higher integrity of RNA and protein cargoes 92. When compared with other isolation methods, such as ultrafiltration or polymer precipitation, SEC offers superior purity by effectively eliminating contaminating protein aggregates and soluble serum proteins. This makes it highly suitable for downstream molecular characterization or therapeutic use.

However, the efficiency of SEC is influenced by column parameters such as pore size and microsphere composition. Improper pore size selection may result in a portion of the protein cargo not being effectively separated or being trapped, affecting recovery. In addition, as the number of column uses increases, the packing may collapse, affecting column efficiency and further reducing recovery. Commonly used SEC gel polymers include crosslinked dextran, agarose, crosslinked polyacrylamide, and polyethylene-stilbene copolymer 93. To address these limitations, researchers have focused on optimizing SEC materials and column conditions. Guo *et al.* 74 for instance, evaluated three types of agarose gels (Sepharose CL-6B, CL-4B, and CL-2B) and identified Sepharose CL-6B as the most effective for serum EV separation. By increasing the column bed volume of CL-6B to 20 mL, they developed a simplified, stable SEC method for rapid separation of high-purity EVs in only two elution steps, saving time and cost.

For enhanced protein cargo recovery, SEC is often combined with immunoaffinity-based approaches. In such workflows, SEC first removes bulk impurities, while subsequent immunocapture specifically isolates exosomes expressing target surface markers, thereby improving both recovery and specificity. Recent innovations such as the SmartSEC system integrate SEC and magnetic bead-based immunocapture, further reducing non-specific binding and improving protein yield.

SEC is ideal for high-resolution exosome isolation due to the small sample size required, ease of operation and compatibility. However, compared to ultracentrifugation and ultrafiltration techniques, SEC requires specialized equipment, columns and packing materials, resulting in higher equipment and consumable costs. Nonetheless, many companies have introduced commercialized exosome separation products based on SEC, which has led to widespread acceptance of the technology in scientific research and clinical applications. However, SEC still faces several challenges in commercialization and scale-up. First, selecting efficient and stable gel materials is critical for large-scale production, as the porosity and stability of the gel directly affects separation efficiency and purity. Second, reducing operating costs and optimizing the process flow are key to achieving economical large-scale production. Finally, scaling up equipment and ensuring stable throughput remain technical challenges to ensure economical and efficient large-scale production.

#### Polymer precipitation-based isolation technology

Polymer precipitation is a widely used technique for exosome isolation, particularly in commercial exosome extraction kits. Among the various polymers, polyethylene glycol (PEG) is the most used reagent. However, PEG-based precipitation struggles to separate exosomes from free proteins and other soluble molecules, which can negatively impact the purity and recovery of exosomes. Additionally, the elution process makes it difficult to completely remove PEG from the exosome surface, potentially compromising the activity of the exosomes and interfering with downstream experiments. A novel solution to these challenges was proposed by Khan *et al.* 94, who introduced a stimulus-mediated exosome enrichment and purification system. This system involves modifying a thermoresponsive, reductant-cleavable copolymer (PNN) onto the phospholipid bilayer of exosomes (PNN-Exos). When the temperature exceeds the low critical phase transition temperature of 31°C, the PNN changes from a hydrophilic to a hydrophobic state, causing the PNN-Exos to spontaneously aggregate. This method yields extremely high exosome purity and recovery, while preserving the biological activity of the exosomes, outperforming traditional methods like ultracentrifugation and PEG-based kits.

In addition, recent studies have explored coupling antibodies or aptamers to polymers to enhance exosome isolation efficiency and purity. This approach simplifies the operational steps, making it more suitable for a wide range of applications and research. For instance, Yu *et al.* 95 developed a CD63 aptamer-modified thermoresponsive polymer (PNB-aptamer) for efficient exosome isolation from MSCs. The PNB-aptamer captures exosomes by introducing sequences complementary to the CD63 aptamer, and allows for gentle release. This method simplifies the separation process for more complex biological samples, supporting the clinical application of exosome-based therapeutics.

In conclusion, the polymer precipitation method, particularly the PEG-based precipitation method, is widely used for exosome isolation due to its ease of operation and low cost. However, challenges remain, including the impact of limited purity and recovery on exosome activity and the difficulty of fully removing PEG residues. Continued research into alternative polymers and modifications to existing methods will be crucial for overcoming these limitations and improving exosome isolation techniques.

### Emerging Innovations in Isolation

#### Immunoaffinity capture-based separation technology

Exosomes are rich in transmembrane proteins (e.g., CD63, CD9 and CD81) and specific biomarker proteins (e.g., EpCAM, HER2 and CA-125) 96, which makes immunoaffinity capture a highly effective strategy for their isolation and enrichment. This method relies on the use of antibodies, aptamers, or other affinity ligands to selectively capture exosomes based on the presence of these surface markers.

Antibodies are the most commonly used recognition tools for immunoaffinity capture, owing to their high affinity and excellent specificity. For instance, Guo *et al.* 97 designed a novel Strep-tag II-based immunomagnetic separation system (SIMI), which modifies the surface of magnetic beads to bind anti-CD63 antibodies via the reversible binding between Strep-Tactin and Strep-tag II. This method efficiently captures exosomes from cell culture supernatants. The experimental results demonstrated that exosomes isolated using this technique maintained their structural integrity and biological activity, highlighting the SIMI system's potential for rapid isolation and enrichment of exosomes. As such, SIMI can serve as a promising new exosome separation tool for applications in disease diagnosis and drug delivery.

Magnetic bead technology, which is frequently employed in immunoaffinity capture, provides excellent superparamagnetism, simple functionalization, and compatibility with various detection strategies, making it ideal for exosome isolation 98. However, conventional immunomagnetic beads have certain limitations, particularly when dealing with samples containing large amounts of non-target substances. These non-target substances may nonspecifically adsorb onto the surface of smooth or rigidly modified beads, which can affect the capture efficiency. To address this issue, Jia *et al.* 99 developed immunomagnetic spiked sphere particles (IMHPs) by introducing nanospike-like structures with redox-responsive PEG fragments and anti-CD63 antibodies at the interface of the magnetic beads. This approach not only improved the capture efficiency of target exosomes (up to 91.7%) but also enabled the negative rejection of impurities, such as cellular debris and larger-sized EVs, through the nanostructuring. The results indicated that IMHPs outperformed the conventional ultracentrifugation method, with a 10-fold increase in purity.

Although antibodies are highly specific and exhibit excellent affinity, their preparation is complex, costly, and unstable. As a result, aptamers—synthetic short-chain nucleic acids—are emerging as an alternative for exosome isolation 100,101. Aptamers offer superior stability, are easy to synthesize, and can be modified flexibly. They also possess the ability to bind to specific target molecules with high affinity in complex samples. For example, Zhang *et al.* 102 constructed a Fe_3_O_4_@TiO_2_-CD63 aptamer with dual affinity and excellent magnetic responsiveness to rapidly isolate exosomes from human urine. In this approach, TiO_2_ binds specifically to the exosome lipid membrane phosphate groups, while the DNA aptamer targets the CD63 protein on the surface of exosomes. Huang *et al.* 103 also designed CD63 aptamers to magnetic nanoparticles Fe_3_O_4_ to achieve simple and efficient exosome isolation.

In recent years, hydrogels have been identified as an excellent carrier for encapsulating and releasing small molecule drugs, biomolecules, and exosomes in a controlled manner. This is due to their biocompatibility, environmental friendliness, and ease of production 104,105. Tang *et al.* 106 designed a DNA hydrogel-based bioseparation system that uses ultra-long single-stranded DNA to recognize and bind CD63-positive exosomes. This system enables non-destructive release of exosomes, preserving their integrity and biological activity. The hydrogel method offers a nondestructive isolation technique that is not only efficient and specific but also preserves exosome integrity, making it an innovative solution for both research and clinical applications.

Moreover, recent studies have suggested that phosphatidylserine (PS), which is highly expressed on exosome surfaces, can also serve as a target for isolation 107. For example, Yoshida *et al.* 108 designed an EV isolation device based on the Ca^2+^-dependent Tim4 protein's specific binding to PS. This method removes the Ca^2+^ chelator to achieve non-destructive release of captured EVs. Zhang *et al.* 109 developed a novel Tim4@ILI-01 immunoaffinity sheet material for exosome enrichment from serum. This strategy is simple, cost-effective, and does not require bulky instruments or sophisticated microfluidic devices, making it an efficient method for exosome capture and isolation.

While the immunoaffinity capture-based isolation method offers high specificity and purity, it is limited by its ability to selectively enrich only those exosomes that express specific surface proteins. As a result, it is difficult to comprehensively capture all exosome subpopulations. Additionally, the elution step in immunoaffinity capture may disrupt the exosome structure, affecting its biological activity and limiting its use in certain high-precision applications. Furthermore, the high cost and complexity of antibody and aptamer preparation, along with potential low yields in large-scale separations, present significant challenges. Reducing costs and improving separation efficiency remain key hurdles for the widespread application of this technology.

#### Microfluidic-based exosome isolation technology

With the increasing depth of exosome research, continuous innovation in separation technology has become crucial. Microfluidic technology has emerged as a cutting-edge method for isolating exosomes due to its high throughput, precise control, and ability to integrate various processes. Microfluidic-based isolation techniques are primarily divided into two categories: one based on immunoaffinity and the other combining microfluidics with acoustic waves, dielectrophoresis (DEP), and other methods to achieve label-free isolation of exosomes based on their electrical and physical properties.

Immunoaffinity-based microfluidic devices utilize specific biomarkers, such as antibodies against CD63, to capture exosomes with high specificity and purity. For example, Kang *et al.* 110 designed a dual-mode immunofiltration device (ExoDIF), which captures exosomes via an anti-CD63 antibody and enables efficient recovery of exosomes through cleavable junction chemistry, allowing for further analysis. Compared to the conventional ExoQuick kit, the ExoDIF device outperforms in terms of high throughput and specificity, enabling efficient capture and release of exosomes. Moreover, Hisey *et al.* 111 engineered an anti-EpCAM-modified herringbone microfluidic device for exosome isolation from high-grade plasma ovarian cancer serum, demonstrating the potential of immunoaffinity isolation for cancer diagnostics. While, Yu *et al.* 112 proposed a highly integrated exosome separation and detection chip that achieves high recovery and purity of exosomes at an injection rate of 4.8 mL/h. Furthermore, based on the high performance of the SIMI system developed by Guo's team 113, they designed a magnetic nanoparticle-based microfluidic chip (ExoCPR) that improves the purity of exosomes by integrating bubble-driven micro-mixers and surface tension-assisted immiscibility filtration technology. The ExoCPR chip demonstrated a capture efficiency of 75.8% and a release efficiency of 62.7%, with exosome purity exceeding 90%. This chip system not only simplifies the process of exosome isolation but also enhances reproducibility and efficiency.

Although these immunoaffinity-based microfluidic systems offer significant advantages in terms of purity and specificity, their complex experimental setup and dependence on specialized equipment remain limiting factors for widespread use. The immunolabeling step is often tedious, complicating the experimental process, while the coupling with equipment such as syringe pumps and fluorescence detectors restricts flexibility. Future research may focus on simplifying labeling steps, improving capture efficiency, and optimizing equipment dependence to enhance the operability and applicability of the technique.

In comparison to immunoaffinity-based methods, label-free exosome isolation techniques have gained significant attention in recent years due to their ability to avoid complex immunolabeling steps. These techniques typically combine microfluidics with acoustic waves, DEP, and other methods to separate exosomes based on their electrical and physical properties. Hua *et al.* 114 developed a microfluidic device based on dual tangential flow filtration to separate and purify exosomes using 200 nm and 30 nm nanoporous membranes. This device offers advantages such as ease of fabrication, low cost, and the elimination of the high pressure and clogging problems commonly encountered with traditional ultrafiltration techniques. Zhao *et al.* 115 introduced an automated exosome purification and assay method that combines a centrifugal microfluidic disk system, functionalized membranes (Exo-CMDS), and a novel aptamer fluorescence system (Exo-AFS). With Exo-CMDS, this platform generates purified exosomes from small blood samples in as little as 8 minutes. It offers low cost, simplicity, high purification rates, reproducibility, and broad applicability. The Exo-AFS detects enriched exosomes by recognizing proteins on the surface, enabling a one-step approach from whole blood injection to exosome isolation and purification. This method is well-suited for low-cost, non-invasive, and routine clinical workflows, making it ideal for early cancer screening.

Acoustic waves combined with microfluidics offer another innovative approach for label-free separation. Surface acoustic waves generate an acoustic radiation force that moves particles in microfluidic channels. Particles of different sizes experience different acoustic forces, enabling size-based separation. Wu *et al.* 116 developed an acoustic-fluidic platform that combines acoustic wave technology and microfluidics to enable label-free, contact-free separation of exosomes directly from whole blood. This platform achieves high purity and yield of exosome isolation through the synergistic action of a cell removal module and an exosome isolation module, offering advantages such as simple operation, speed, efficiency, and biocompatibility. Naquin *et al.* 117 introduced the ASCENDx platform, which uses microacoustic wave technology and a rotating droplet system to efficiently purify exosomes and rapidly detect miRNA biomarkers. By optimizing the disc channel geometry to enhance sample enrichment, the ASCENDx liquid biopsy platform shows great potential for liquid biopsy analysis. This platform overcomes the shortcomings of traditional exosome processing and subsequent miRNA detection, providing a promising new solution for biomedical research and diagnostic applications.

DEP is another effective label-free separation technique. It operates by applying force in a non-uniform electric field, exerting varying forces on exosomes based on the electric field strength 80,118. Niu *et al.* 119 proposed a multistage continuous DEP isolation microfluidic chip that increases the lateral displacement of target particles by arranging electrodes and creating a gradient electric field. This approach improves the purity, efficiency, and stability of the isolation system. While DEP faces challenges such as low purity and resolution, advancements in chip design and optimization of electric field parameters have led to significant progress.

The application of microfluidics in exosome isolation, particularly in combination with innovative label-free technologies, demonstrates great potential for future development. Label-free separation techniques have important advantages, including simplified operation and reduced experimental errors. However, challenges remain in achieving high separation efficiency and precision. Continued advancements in microfluidic design and optimization of separation techniques will likely address these issues and expand the range of potential applications for exosome isolation.

### Challenges in Exosome Isolation for Clinical Translation

The clinical translation of exosomes involves the need for large-scale production, making industrialization a critical focus. Among the various isolation techniques, some show strong potential for industrial application. For example, SEC is widely accessible, is easy to operate, and adaptable to diverse sample types. Immunoaffinity-based methods offer high specificity and precision, making them particularly promising for applications in precision medicine and disease biomarker detection. Ultrafiltration stands out as a convenient, cost-effective, and efficient approach, capable of high-throughput processing and rapid isolation of high-quality exosomes, thus suitable for large-scale clinical screening. In contrast, traditional ultracentrifugation and emerging microfluidic methods face significant hurdles in scaling for industrial production. Ultracentrifugation, though prevalent in laboratory research, requires complex operation and specialized equipment, limiting its feasibility for large-scale clinical production.

It is worth noting that although progress has been made in isolating and purifying exosomes using the methods described above, the exosomes obtained still contain many impurities, which can adversely affect exosome bioactivity and therapeutic efficacy. Regulatory agencies, including the Food and Drug Administration (FDA), have stringent requirements for the purity, potency, safety and efficacy of exosome products to ensure that they can be approved for clinical use. Therefore, comprehensive characterization and rigorous quality control to ensure high purity are essential 120. To meet regulatory requirements, a comprehensive characterization of exosomes is necessary. Table 4 summarizes key parameters and corresponding methods for exosome characterization.

NTA is a commonly used measurement in EV characterization to estimate the overall size and concentration of particles in solution. However, as NTA analyzes all particles in suspension collectively based on light scattering, it cannot distinguish exosomes from other non-exosomal particles. Therefore, it is recommended to complement NTA with antibody-based techniques or high-resolution imaging (e.g., TEM or SEM) for accurate identification. In contrast, NanoFCM enables single-particle detection based on light scattering and multicolor fluorescence, allowing for simultaneous analysis of size, surface marker expression, and other physicochemical properties. This provides a more detailed and quantitative characterization of EVs.

It should be noted that different characterization methods and equipment may lead to differences in detection results. Therefore, for accurate characterization of individual EVs, a combination of at least two complementary methods (e.g., SEM/TEM in conjunction with NTA) is usually required for confirmation 120. Regarding the identification of EV markers, there is currently no universal identifier for EVs due to the varied composition of EVs from different sources. The selection of markers depends on the source and type of sample.

Commonly used markers for exosome surface marker identification include tetratransmembrane proteins (e.g., CD9, CD63, and CD81) and late endosomal-associated proteins (e.g., Tsg101 and Alix) 121. Following the guidelines of the International Society for Extracellular Vesicles (ISEV), exosome characterization requires the detection of at least three protein markers, typically two positive markers and one negative marker, to ensure specificity. This is most often achieved through immunoblotting techniques 122. Finally, the purity of exosome preparations can be evaluated through various approaches, with the most widely used index being the ratio of particle number to total protein content.

In conclusion, although existing isolation and characterization techniques have provided strong support for exosome research, they still face many technical and standardization challenges during industrial production and clinical translation. Future research should focus on optimizing the existing techniques to enhance the separation efficiency and purity, as well as strengthening the standardization of separation and characterization techniques to promote the application of exosomes in clinical therapy.

### Good Manufacturing Practices (GMP) Considerations and Cost Challenges

Among the biggest challenges in advancing exosome-based therapies into clinical applications, especially in the field of NDDs, is achieving scale-up production that meets the requirements of Good Manufacturing Practices (GMPs). GMPs are essential for ensuring product quality, safety, and reproducibility, but they also introduce considerable costs and technical challenges. Key issues in exosome GMP production include upstream cell culture, downstream purification, and comprehensive quality control 123.

In the upstream cell culture process, the choice of culture medium is a primary cost driver. It is also crucial for minimizing risks such as immune reactions and viral contamination. Culture media are broadly categorized into serum-containing and serum-free formulations. Although serum-free media are significantly more expensive, more complex to formulate, and yield lower initial production volumes, they offer important long-term advantages for GMP-compliant manufacturing. These advantages include improved batch-to-batch consistency, the elimination of animal-derived pathogens, and easier regulatory approval—factors that collectively reduce the likelihood of batch failures, safety concerns, and regulatory delays. Conversely, traditional serum-containing media may seem cost-effective initially but are associated with higher risks of batch variability, contamination, and regulatory hurdles that can ultimately increase overall production costs and impede scale-up. Thus, serum-free media are increasingly recognized as a more sustainable and compliant choice for large-scale, clinical-grade exosome production.

In the downstream purification process, achieving GMP-grade exosome purity involves efficient removal of host cell proteins, DNA contaminants, and non-target EV populations. Traditional manual methods such as ultracentrifugation, though widely used in research settings, are labor-intensive, low-throughput, and difficult to standardize at industrial scale. In contrast, automated purification technologies—such as TFF, SEC, and novel systems like EXODUS—require significant upfront investment but offer superior scalability, improved reproducibility, reduced labor demands, and lower contamination risks. These technologies are essential for enabling consistent and efficient large-scale production.

In conclusion, the clinical translation of exosome-based therapies must proactively address the high cost and complexity of GMP-compliant manufacturing. Establishing standardized production workflows—centered around serum-free culture systems and automated purification platforms—and continually optimizing yield and cost-efficiency are critical steps toward overcoming scalability bottlenecks. These advances are fundamental for making exosome therapeutics accessible to a broader population of patients with NDDs.

### Innovations in Exosome Drug-Carrying Technologies

Exosome-based drug delivery has emerged as a promising method for targeted therapy, utilizing the unique properties of exosomes to transport a wide range of therapeutic molecules. The drug loading into exosomes can be categorized into two main techniques: endogenous loading and exogenous loading. These technologies enable the delivery of various therapeutic agents, offering a versatile platform for treating neurological disorders and other diseases.

#### Endogenous loading technology

Endogenous loading technology involves bioengineering donor cells to produce exosomes that contain specific drugs or molecules. In this process, target molecules are introduced into donor cells through methods such as direct transfection or co-incubation. Endogenous loading techniques can be applied to various therapeutic molecules, including RNA, proteins, and peptides. Below, we briefly outline the latest research advancements in the genetic engineering of exosome drug loading.

Currently, most studies focus on the loading of nucleic acids into exosomes via the transfection of RNA-coding plasmids. A notable breakthrough by Zickler *et al.* 124 developed an endogenous loading platform that significantly improves both the loading and delivery efficiency of exosomes. They engineered cells to stably express target mRNAs by fusing CD63 with the high-affinity RNA-binding protein PUF, resulting in exosomes loaded with mRNA. This platform addresses several limitations of existing endogenous loading methods, such as low mRNA loading efficiency, poor delivery rates, and interference from plasmid DNA in mRNA quantification and functional analysis. Additionally, Fu *et al.* 125 designed artificial genetic circuits to reprogram cells, enabling them to direct the self-assembly of exogenous small interfering RNAs (siRNAs) into secreted exosomes and facilitating systemic or targeted delivery of these self-assembled siRNAs *in vivo*.

Another innovative approach by Gu *et al.* 126 utilized an mRNA delivery nanocarrier targeting brain neuronal cells by transfecting leukocytes with DNA or RNA encoding the virus-like protein capsid Arc, along with the Arc-5'UTR motif (A5U). The Arc protein self-assembles into a virus-like capsid, encapsulating and releasing the mRNA within neuronal cells. Their study showed that incorporating Arc increased mRNA loading by over sixfold, and adding the A5U motif further stabilized the Arc capsid, enhancing the mRNA loading efficiency. This breakthrough offers new prospects for treating neurodegenerative diseases and opens fresh research directions in biomedical engineering. Despite success in chronic low-grade neuroinflammation models, further validation of the loading and delivery efficiency is necessary for acute or more severe neuroinflammatory conditions.

Protein therapeutics play a crucial role in maintaining normal body functions. Traditional protein delivery methods, such as electroporation and mechanical force-induced temporary membrane openings, are effective but complicated, costly, and can disrupt membrane integrity, potentially affecting normal cellular function. In contrast, exosome-based drug delivery systems show greater promise as efficient nanocarriers for therapeutic proteins. Han *et al.* 127 developed the MAPLEX system, a novel engineered exosome technology that utilizes photocleavable proteins (mMaple3) for controlled protein release. This system enables the regulation of various complex cellular events, including transcription, gene recombination, and epigenome editing. The MAPLEX exosome is created by coupling the N-terminus of the exosome scaffold protein CD9 with mMaple3. The team used this system to deliver dCas9-D3A into an AD mouse model, inducing methylation at a target CpG site, which reduced Bace1 expression, amyloid pathology, and memory impairment. Compared to Kong *et al.* 128 who used a different delivery method, the intranasal delivery in the MAPLEX system proved to be more efficient, safer, easier to administer, and demonstrated higher bioavailability and broader applicability. This approach offers a novel strategy to address protein delivery challenges in NDDs and opens up new possibilities for gene therapy. However, since the MAPLEX system lacks a targeting component, further research is needed to integrate appropriate targeting mechanisms and select the right exosome-producing cell types to enhance targeting specificity.

One of the main advantages of endogenous loading techniques is their ability to minimize disruption to exosome membranes, preserving their natural structure and improving biocompatibility and stability. This makes exosomes a highly promising drug delivery system. However, this method remains relatively complex and costly, requiring further optimization to enhance productivity and reduce operational costs.

#### Exogenous loading technology

Exogenous loading techniques involve loading drugs onto the surface or inside exosomes that are isolated and purified from cell cultures or other biological fluids, using physical or chemical methods. Common loading approaches, such as electroporation, sonication, freeze-thawing, saponification, extrusion, and co-incubation, have been widely studied (Table 5). This section will focus on recent advancements in the chemical modification of exosome surfaces.

Surface chemical modification enhances the drug-loading capacity of exosomes by attaching specific molecules (e.g., peptides, ligands, aptamers) to their surface. This approach not only increases the functionality of exosomes but also circumvents the limitations of traditional drug-loading methods (e.g., transfection, electroporation, freeze-thawing), which often result in poor loading efficiency. A common surface modification strategy involves anchoring peptides, such as the CP05 peptide. CP05 has a high affinity for the exosome surface protein CD63, and its attachment significantly improves drug-loading efficiency. For instance, Gao *et al.* 138 demonstrated that anchoring CP05 to the exosome surface enhanced its drug-loading capacity. Xu *et al.* 139 employed a similar strategy, coupling a cell-penetrating peptide (R9 peptide) to the exosome surface through an amide bond, enabling the loading of antisense oligonucleotides (ASOs). The addition of the R9 peptide simplifies the loading process, making it more suitable for large-scale production of functionalized exosomes and providing a novel design for efficient drug delivery systems.

In recent years, surface modification methods for loading nucleic acid drugs have gained traction. One common strategy involves inserting cholesterol or CP05 peptides into the membrane phospholipid bilayers to load nucleic acids 140. Another promising method is the use of aptamers, which can load nucleic acid drugs onto exosomes through direct synthesis or annealing. This strategy bypasses the complexity and high costs associated with traditional peptide-nucleic acid coupling. Han *et al.* 141 used the systematic evolution of exponentially enriched ligands (SELEX) technique to identify a DNA aptamer that efficiently loads various nucleic acid drugs onto the exosome surface. This simple and scalable approach holds great potential for advancing exosome-based nucleic acid therapies, potentially making oligonucleotide therapies more effective and cost-efficient.

Additionally, the combination of metabolic engineering and click chemistry has introduced new opportunities for surface modification of exosomes. Bhatta *et al.* 142 designed a versatile exosome loading platform using a metabolic engineering approach. They enabled parental cells to uptake Ac4ManAz (tetraacetyl N-azidoacetylmannosamine), which added azide groups to the exosome surface. Click chemistry was then employed to efficiently load drug molecules onto the exosomes. This strategy not only simplifies the surface modification process but also improves the efficiency and diversity of drug loading, expanding the potential applications of exosomes in drug delivery.

## Engineered Exosome Strategies for Enhanced Brain Targeting and Regulation of Signaling Pathways

### Targeted engineering of exosomes for brain delivery

**Figure 4:**
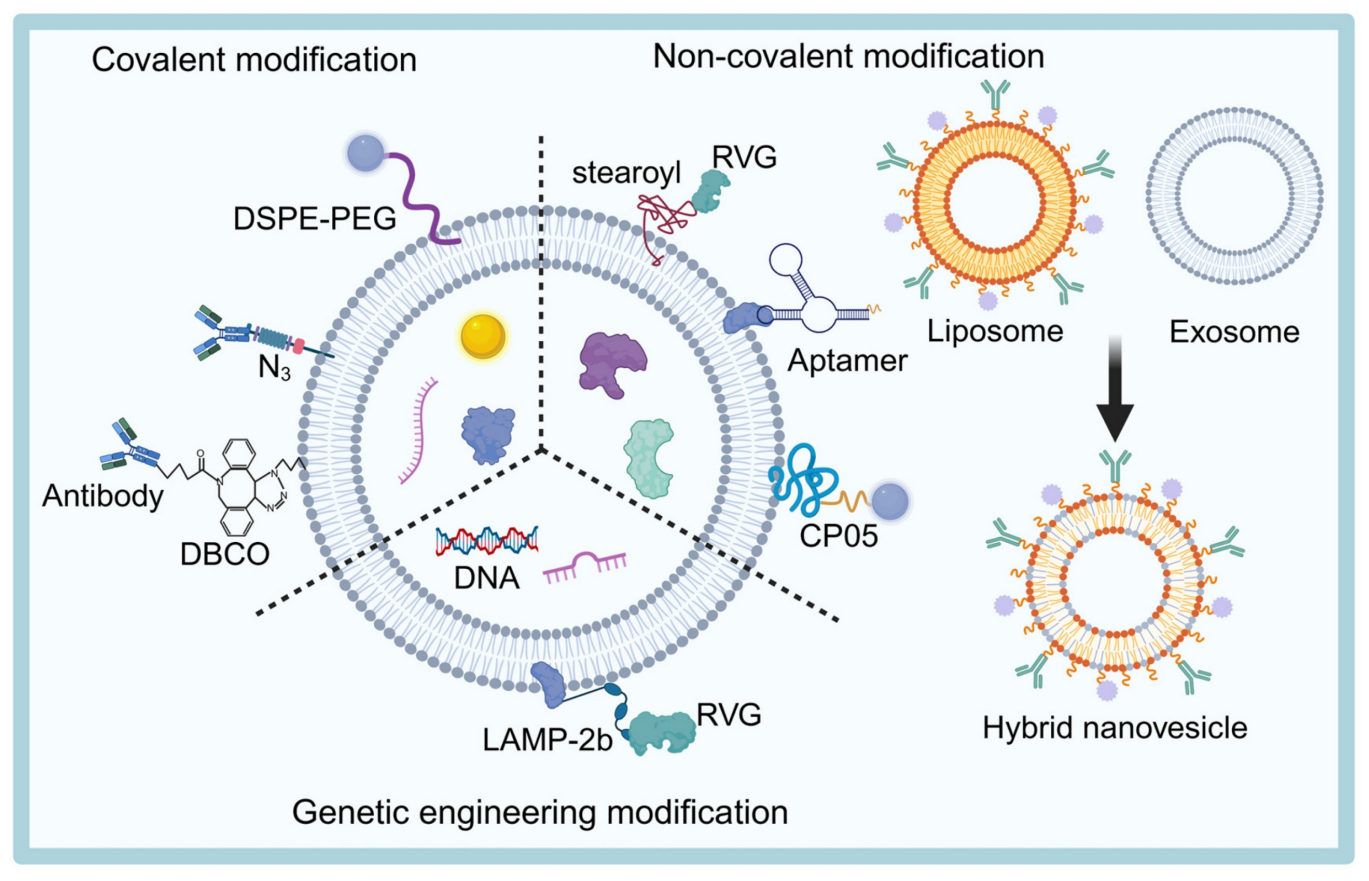
** Schematic representation of exosome surface modification to enhance brain targeting.** Covalent modification involves stable chemical conjugation techniques, while non-covalent modification relies on reversible interactions through hydrophobic insertion. Genetic engineering approaches modify parent cells to express membrane-anchored targeting peptides. The figure was created with BioRender.

Exosomes are multifunctional nanoscale vesicles that exhibit significant diversity and are rich in surface proteins. However, due to the lack of clear tissue specificity, the targeting potential of exosomes is not inherently high. To address this, researchers frequently employ surface modification techniques to enhance exosome targeting, stability, and delivery capabilities. Below, we summarize some of the current strategies commonly used to enhance the brain-targeting ability of exosomes (Figure 4).

#### Genetic engineering modification

Genetic engineering of donor cells or exosomes is a widely used surface modification technique. The process involves constructing a fusion plasmid that links a target protein with an exosome scaffolding protein, which is then transfected into parental cells. This allows the generation of stable monoclonal cell lines that express the target protein, facilitating large-scale production of engineered exosomes 143. The surface of these modified exosomes often expresses specific targeting sequences, peptides, or antibodies, enabling precise delivery to specific cells or tissues 144,145.

Common exosome scaffolding proteins include type I transmembrane proteins (e.g., Lamp2), tetraspanning proteins, and peripheral membrane proteins (e.g., MFGE8) 146. Among these, Lamp2b is the most utilized exosome scaffolding protein. The N-terminal of Lamp2b is located outside the exosome membrane, allowing it to strongly fuse with specific targeting sequences or peptides to construct a targeted drug delivery system 147,148. For example, Alvarez-Erviti *et al.* 129 successfully created brain-targeted exosomes (RVG-Exo) by modifying dendritic cells to express Lamp2b, fused with a peptide fragment from the neuron-specific rabies virus glycoprotein (RVG). Intravenous injection of RVG-Exo enabled drug-specific delivery to neurons, microglia, and oligodendrocytes in the brain. Similarly, Yang *et al.* 149 co-transfected HEK293 cells with RVG-Lamp2b and a nerve growth factor (NGF) plasmid, generating exosomes that efficiently delivered NGF to the brain. Jiang *et al.* 150 further optimized exosome brain targeting by modifying the surface with three BBB shuttle peptides (RVG29, TAT, or Ang2). The results indicated that RVG29-modified exosomes exhibited superior targeting efficiency to the brain and could serve as stable brain-targeted drug delivery vehicles.

In addition to RVG, other peptides have been explored to further enhance exosome brain targeting. One such peptide is angiopep-2, a Kunitz-type peptidase inhibitor with high affinity for the low-density lipoprotein receptor-related protein 1, known for its excellent brain penetration capability 151. For instance, Zhu *et al.* 152 developed a bipeptide-modified small EVs drug delivery system, combining the targeting properties of angiopep-2 with the membrane-penetrating properties of TAT, enabling efficient targeting of chemotherapeutic drugs to the mouse brain. Moreover, the fusion of the T7 peptide with Lamp2b has been shown to further improve exosome brain targeting and enhance their ability to traverse the BBB 153.

However, current exosome scaffolding proteins do not fully meet the diverse requirements of engineered modifications. Therefore, identifying novel scaffolding proteins is of great importance. Recently, Zhao *et al.* 154 introduced a new screening criterion for exosome scaffolding proteins and identified PLXNA1 as a promising candidate with excellent sorting capability. This discovery opens new possibilities for engineering exosomes with enhanced delivery properties.

The advantages of genetically engineered exosomes include enhanced drug delivery efficiency, the ability to cross the blood-brain barrier, improved drug stability and biocompatibility, prolonged circulation time, and the capacity to carry multiple therapeutic molecules. However, there are also several drawbacks to genetic modification. These include technical complexity and high costs, which may result in a loss of bioactivity and potential immune responses. Additionally, challenges related to production efficiency and purity could impact the clinical applicability of these engineered exosomes.

#### Covalent modification

In addition to genetic engineering, covalent modification is a widely used technique for modifying the surface of exosomes. Through methods like click chemistry or azide-alkyne cycloaddition reactions 155, targeting ligands can be covalently attached to the exosome membrane, thereby enhancing the targeting ability of the exosome.

A notable example of this approach is the work of Sul *et al.* 156, who successfully bound dopamine to the surface of adipose stem-cell-derived EVs (Dopa-EVs) using a simple and direct cross-linking reaction, enabling selective targeting of dopaminergic neurons in PD. By co-culturing the exosome with neurons, it was found that dopamine enhanced the uptake of Dopa-EVs by neurons through dopamine receptor-mediated pathways. Importantly, this modification did not significantly alter the basic physical and biological properties of the exosomes, highlighting their potential for application in neurodegenerative diseases. Additionally, Zhang *et al.* 157 engineered a multi-targeted exosome (MP@Cur-MExo) derived from activated neutrophils. This exosome effectively targets activated neurons in AD brains by conjugating superparamagnetic iron oxide nanoparticles modified with both mitochondria-targeting and β-amyloid (Aβ)-targeting ligands via click chemistry, facilitating multi-targeted therapy.

Click chemistry offers a straightforward and efficient approach for exosome functionalization. However, there are some limitations to this method. Since exosome membranes contain various membrane proteins, covalent modifications may interfere with the function of these proteins, potentially affecting the exosome's biological properties and delivery capabilities. Therefore, precise control of the reaction conditions is essential to ensure that the targeting ligand binds to the exosome membrane without disrupting the natural function of the exosome or diminishing its biological activity.

#### Non-covalent modification

Non-covalent modification is another commonly used strategy for exosome surface modification. Liu *et al.* 158 designed a REXO-coated gene-chemical nanocomplex that efficiently crosses the BBB, targets neurons, and releases drugs in the high-reactive oxygen species (ROS) environment of dopaminergic neuronal lesions. They achieved this by embedding stearoyl-RVG within the phospholipid bilayer of exosomes through non-covalent interactions, enhancing their targeting and delivery capabilities. This method offers a gentle approach for modifying exosome surface properties, ensuring high stability and biocompatibility while preserving the exosome's natural state.

Another novel non-covalent modification method involves the hydrophobic interaction between liposomes and exosomes 159. Sato *et al.* 160 introduced a fusion technique, where exosome and liposome membranes are combined via a freeze-thaw process to create engineered hybrid exosomes. These hybrid exosomes exhibit improved delivery functionality and optimized surface properties. Furthermore, Jiang *et al.* 161 developed ROS-responsive mimetic exosome-liposome hybrid nanovesicles by fusing angiopep-2-modified exosomes with liposomes. These hybrid exosomes effectively target the BBB and enhance drug accumulation at AD lesions, ultimately improving cognitive function.

Compared to covalent modifications, non-covalent modifications have a lesser impact on the natural structure of exosomes, offering higher stability and biocompatibility. Additionally, they are more cost-effective and provide greater flexibility. Although non-covalent modifications are milder, they may not be as stable as covalent modifications, especially during long-term storage or *in vivo* circulation, which could lead to the separation or failure of the modifiers.

#### Innovative strategies

Most existing engineering methods modify exosomes as a whole, which can compromise the integrity of the membrane structure and the activity of surface proteins or nucleic acids, resulting in impaired targeting and therapeutic efficacy 162. Moreover, the strategy of engineering donor cells to produce specific exosomes involves cumbersome steps, high costs, and limited controllability.

To address these challenges, researchers have proposed innovative approaches. For instance, Wang *et al.* 163 combined nanoscale artificial modules with specific functions (nitric oxide (NO)-driven nanomotors capable of chemotaxis in PD microenvironments with high expression of iNOS/ROS) with natural exosome modules in a “one-to-one” fashion. This created engineered exosomes with “stand-alone module/cascade functions.” Thanks to the unique transmembrane ability of the natural exosome module, the artificial module drives the exosome to efficiently pass through the BBB, precisely target damaged neuronal cells and mitochondria in the disease microenvironment, and effectively treat PD through multi-step interventions. This approach reduces the non-specific binding typically seen in traditional engineered exosomes within the disease microenvironment, offering an excellent solution for enhancing targeting ability after crossing the BBB.

Surface modification technology for exosomes holds great potential in brain-targeted drug delivery. Genetically engineered modifications, covalent modifications, and non-covalent modifications each have their respective advantages and limitations. The choice of the most appropriate strategy depends on the specific goals and requirements of the application. With ongoing advancements in technology, more novel modification methods are likely to emerge, further enhancing the potential of exosomes in drug delivery and disease treatment.

### Exosome-Mediated Regulation of Signaling Pathways

**Figure 5:**
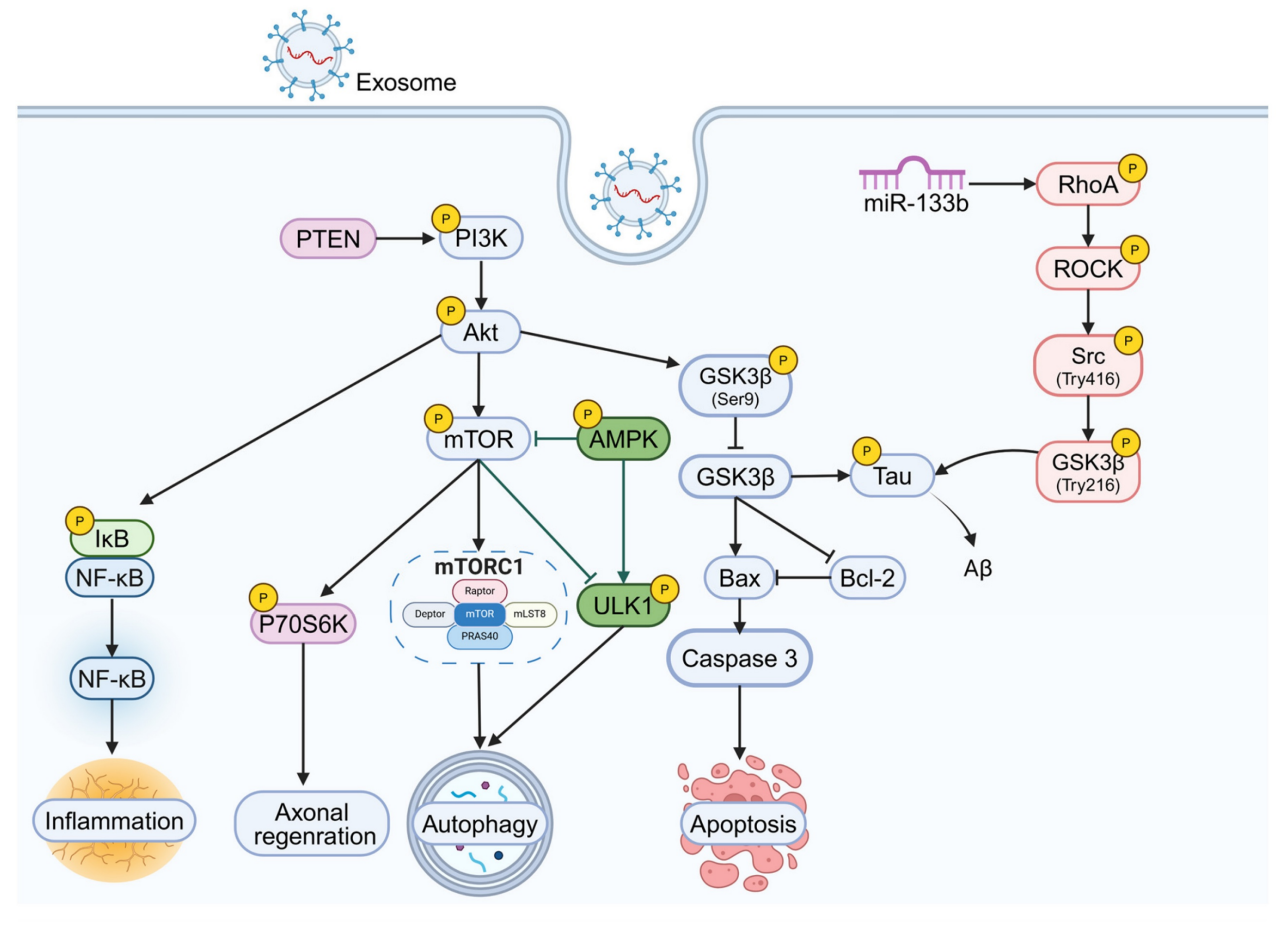
** Schematic diagram of exosome-mediated treatment of NDDs via targeted signaling pathways.** PI3K: phosphatidylinositol3 kinase; Akt: protein kinase B, PKB; GSK-3β: glycogen synthase kinase-3β; mTOR: mechanistic target of rapamycin; NF-κB: Nuclear factor kappa-B; Tau: tau protein; PTEN: phosphate and tensin homology deleted on chromosome ten; AMPK: adenosine 5'monophosphate-activated protein kinase; Bcl-2: B-cell lymphoma 2; Bax: bcl-2-associated X protein; ULK1: unc-51 like autophagy activating kinase 1; P70S6K: p70 ribosomal protein S6 kinase; IκB: Inhibitor of NF-κB; RhoA: Ras homolog gene family member A; ROCK: Rho-associated coiled-coil-containing protein kinase. The figure was created with BioRender.

Exosomes play a pivotal role in regulating intracellular signaling pathways in neurons, which is crucial for the pathophysiology of NDDs. Several key signaling pathways, including PI3K/Akt/GSK-3β, PI3K/Akt/mTOR, PI3K/Akt/NF-κB, and RhoA-ROCK, are involved in neuronal survival, apoptosis, autophagy, and neuroinflammation 164-166 (Figure 5). In addition, it has been shown that human endometrial stem cells-derived small EVs can be used to treat nerve injury by activating the PI3k/AKT signaling pathway to enhance cell proliferation and migration and promote nerve growth 167. Here we briefly describe how exosomes regulate these pathways and their potential therapeutic role in NDDs.

#### PI3K/Akt/GSK-3β signaling pathway

GSK-3β is widely expressed in the central nervous system and is a key participant in normal brain function signaling 168. Research has shown that the PI3K/Akt/GSK-3β signaling pathway is closely linked to neuronal apoptosis 169. In this pathway, Akt phosphorylates a specific site on GSK-3β (Ser9), inhibiting its activity and preventing further phosphorylation of β-catenin 170. In PD, GSK-3β activation increases the expression of the pro-apoptotic protein caspase-3, promoting neuronal death 171. The PI3K/Akt/GSK-3β pathway is not only crucial for neuronal survival but also regulates Tau protein phosphorylation, which is a key mechanism in AD 172. Overactivation of GSK-3β leads to excessive Tau phosphorylation 173. In AD, exosomes carrying curcumin can activate the PI3K/Akt/GSK-3β pathway, inhibiting Tau phosphorylation and preventing neuronal death, both *in vitro* and *in vivo*, thereby alleviating AD symptoms 137.

#### PI3K/Akt/mTOR signaling pathway

The mTOR is a key downstream target of the PI3K/Akt pathway and a critical autophagy regulator 174. Upon Akt activation, mTORC1 is phosphorylated, enhancing protein translation and inhibiting autophagy 175. The PI3K/Akt/mTOR pathway is essential for autophagy regulation, which is a key process in maintaining neuronal function 176.

Exosomes have been shown to modulate this pathway to enhance autophagic flux and promote neuroprotection. For example, in AD mouse models, rapamycin, a selective mTOR inhibitor delivered via exosomes, reduces mTOR activation, increases microtubule-associated protein 1 light chain 3-II (LC3-II) expression, and promotes neuronal autophagy 177. Ebrahim *et al.* 178 assessed the therapeutic mechanism of MSCs-Exos in AD and analyzed the complex relationship between the PI3K/AKT/mTOR pathway and the regulation of autophagy. Liu *et al.* 179 found that adipose stem cell exosomes can activate mitochondrial autophagy through inhibition of the PI3K/AKT/mTOR pathway.

#### PI3K/Akt/NF-κB signaling pathway

NF-κB (nuclear factor kappa-light-chain-enhancer of activated B cells) is a transcription factor that, in its inactive state, is bound to IκB, limiting its movement in the cytoplasm 180. Akt can phosphorylate IκB kinase, leading to IκB degradation and the release of NF-κB, which translocates to the nucleus to activate the expression of inflammatory genes such as tumor necrosis factor (TNF)-α and IL-1β 181. In NDDs such as AD and PD, abnormal activation of the PI3K/Akt/NF-κB pathway is associated with increased inflammation 180. Lee *et al.* 182 found that exosomes containing non-degradable IκB (Exo-srIκB) can inhibit NF-κB activation, thereby alleviating neuroinflammation. Exosomes carrying miR-124-3p have been shown to suppress neuroinflammation by modulating the MYH9/PI3K/Akt/NF-κB signaling cascade in neurons 183. Additionally, MSC-Exos can regulate inflammation through the STAT3-NF-κB pathway in APP/PS1 mouse models, improving cognitive function 184.

#### RhoA-ROCK signaling pathway

RhoA, a small GTPase in the Rho family, is a key regulator of the cytoskeleton and cell adhesion dynamics, with its main downstream effectors being the ROCK1/2 serine/threonine kinases 185. Activation of the RhoA-ROCK pathway promotes cell death and mediates the loss and retraction of synapses and neuronal pathways in the brain. Inhibiting RhoA-ROCK signaling is considered a promising strategy for treating CNS diseases 186. Numerous studies have shown that inhibiting RhoA-ROCK signaling benefits the treatment of ALS, PD, and AD. For example, RVG29-Exo-133b, which targets the RhoA-ROCK pathway, reduces Tau phosphorylation and enhances motor function, mitigating neurodegeneration in PD models 150. Schwann cell-derived exosomes targeting the RhoA-ROCK pathway have also been shown to reduce activation of protein tyrosine phosphatase sigma and promote axon regeneration following spinal cord injury 187.

#### Other signaling pathways

In addition to the aforementioned pathways, other signaling cascades are crucial in NDDs. For instance, the AMPK/mTOR pathway plays a significant role in regulating autophagy. Exosomes derived from human umbilical MSCs (hUMSC-Exos) have been shown to activate this pathway, enhancing autophagy and promoting spinal cord endothelial cell repair 188. Furthermore, PTEN, a negative regulator of the mTOR pathway, is involved in axon regeneration and neuronal survival 189,190. MSC-Exos loaded with miR-26a can activate the PTEN-AKT-mTOR pathway, promoting axon regeneration and neurogenesis 191. Additionally, miR-146a-5p from hUMSC-Exos reduces microglia-mediated neuroinflammation through the IRAK1/TRAF6 pathway, contributing to the attenuation of neurodegenerative processes 192.

By targeting these pathways, exosomes hold significant therapeutic potential for treating neurodegenerative diseases. Despite challenges in optimizing exosome-based therapies, their ability to modulate critical signaling pathways highlights their promise as effective treatments for NDDs. Further research is needed to fully explore the therapeutic potential of exosome-based interventions in clinical applications.

## Exosome-Based Therapies for NDDs

Exosomes have gained significant attention in the treatment of NDDs due to their versatility and potential to address multiple challenges in the nervous system. In particular, AD and PD are major targets for exosome-based therapeutic strategies. Researchers are investigating the use of exosomes to deliver neuroprotective factors, clear Aβ, and repair damaged neurons, with the goal of slowing disease progression and improving clinical symptoms. Additionally, exosomes have shown potential in promoting nerve regeneration and restoring cognitive function, offering new hope to patients with NDDs.

### Therapeutic Potential in NDDs

#### Alzheimer's disease (AD)

AD is a neurodegenerative disorder primarily affecting middle-aged and older adults, characterized by cognitive decline. The pathological features of AD include the accumulation of β-amyloid plaques and neurofibrillary tangles formed by P-Tau. These hallmark features contribute to the neuronal damage observed in the disease. Despite its devastating impact, current pharmacological treatments for AD are limited to cholinesterase inhibitors and NMDA antagonists, which primarily address symptoms rather than underlying causes. The main barrier to effective treatment is the challenge of delivering drugs across the BBB and the pharmacokinetic limitations of available therapies.

Recent research has focused on targeting Aβ and P-Tau accumulation as a therapeutic strategy for AD. One promising approach involves the use of enkephalinase, an enzyme that degrades Aβ and reduces its pathological accumulation. Katsuda *et al.* 193 demonstrated that human adipose tissue-derived MSCs (ADSCs) secrete exosomes containing functional enkephalinase. *In vitro* experiments showed that ADSC-derived exosomes (ADSC-Exos) could be internalized by neuroblastoma cells, resulting in a reduction of intracellular Aβ levels. These findings suggest that ADSC-Exos could serve as a promising drug delivery vehicle for Aβ clearance in AD. Further supporting this idea, Elia *et al.* 194 injected MSC-Exos containing enkephalinase into the cerebral cortex of mice, which led to a reduction in Aβ plaque formation in the hippocampus and cerebral cortex, as well as a decrease in dystrophic neurite formation.

With in-depth studies on the pathogenesis of AD, researchers have found that exosomes can induce autophagy, thereby clearing misfolded proteins in NDDs, including AD 195. In this context, Iyaswamy *et al.* 196 engineered exosomes by modifying Fe65, a protein that interacts with amyloid β precursor protein (APP), onto their surface to create a novel targeted drug delivery system, Fe65-Exos. This system was designed to precisely deliver the autophagy inducer Corynoxine-B (Cory-B) to neurons with high APP expression. By analyzing the fluorescence intensity and co-expression of Fe65 and APP in the mouse brain, it was demonstrated that Fe65-Exos could effectively cross the BBB and deliver Cory-B to the brains of AD mice. *In vitro* and *in vivo* experiments further showed that Fe65-Exos-Cory-B enhanced BECN1-dependent autophagy in neurons and promoted synaptic plasticity formation among hippocampal neurons in AD mice, thus improving learning and memory deficits in 5xFAD mice. This study provides an innovative exosome-based drug delivery system with AD-specific targeting, offering a new strategy for the future treatment of AD and other NDDs. However, further studies are needed to explore the potential side effects of this system and to develop an effective intranasal delivery method.

Beyond Aβ and tau pathology, AD is also associated with neuroinflammation, oxidative stress, and mitochondrial dysfunction 197. Neuroinflammation, driven by the activation of astrocytes and microglia, is a key feature of AD and many other NDDs. Modulating neuroinflammation could therefore provide an effective therapeutic strategy. RVG-modified MSC-Exos have been shown to attenuate plaque deposition, reduce Aβ accumulation, and inhibit astrocyte activation, while also downregulating pro-inflammatory cytokines (such as TNF-α, IL-1β, and IL-6) and upregulating anti-inflammatory factors (such as IL-4 and IL-10) 198. These effects led to improvements in spatial learning and cognitive function in AD mice.

Moreover, NSC-Exos have demonstrated the ability to reduce P-Tau levels and Aβ formation by inhibiting kinase activity, thereby downregulating the NF-κB/ERK/JNK signaling pathway in activated microglial cells. In preclinical models, NSC-Exos have been shown to effectively mitigate the pathogenesis of AD by reducing neuroinflammation and promoting neuronal health 94. Human umbilical cord MSC-derived exosomes (hucMSC-Exos) have also been reported to modulate microglial activation and reduce Aβ deposition, improving spatial learning and memory in AD mice 199.

In addition to their anti-inflammatory and neuroprotective effects, MSC-Exos are capable of protecting against oxidative stress and synaptic damage caused by Aβ toxicity, further supporting their potential as a therapeutic tool for AD 200. Huber *et al.* 201 showed that exosomes secreted by neural stem cells in response to heat shock stimulation exhibit enhanced neuroprotection against oxidative stress and Aβ-induced neurotoxicity. Mitochondrial dysfunction is another key contributor to AD, as it leads to increased ROS, oxidative damage, and synaptic defects. Targeting mitochondrial dysfunction offers a promising therapeutic avenue. NSC-Exos have been found to activate the SIRT1-PGC1α signaling pathway, enhancing mitochondrial function, restoring mitochondrial biogenesis markers (such as PGC1α, COXIV, and NRF1), and improving cognitive function in AD mice 202. Additionally, Zhang *et al.* 157 designed a multi-target engineered activated neutrophil exosome (MP@Cur-MExo), which improves mitochondrial health by inhibiting extracellular Aβ deposition and aggregation, as well as mitochondrial autophagy. This approach attenuates Aβ-induced mitochondrial metabolic defects, providing a promising new direction for AD therapy.

#### Parkinson's disease (PD)

PD is a common neurodegenerative disorder that manifests in both motor (myotonia, resting tremor, bradykinesia) and non-motor symptoms (cognitive/psychiatric abnormalities, autonomic dysfunction, sensory deficits). Pathologically, PD is characterized by the degeneration and death of dopaminergic neurons in the substantia nigra, along with abnormal deposition of amyloid fibrils (α-syn aggregates forming Lewy bodies) 203,204.

Recent studies have revealed that the spread of α-syn between neurons accelerates the progression of PD 205. For instance, Mao *et al.* 206 found that binding of α-syn preformed fibrils (PFF) to lymphocyte activation gene 3 (LAG3) can trigger endocytosis, delivery and its toxicity of α-syn PFF. As a result, targeting the propagation of pathological α-syn is emerging as a promising therapeutic strategy for PD treatment 207. Exosomes are key mediators of the pathological progression of PD by facilitating the transfer of α-syn between neurons 208,209. Xia *et al.* 210 injected exosomes derived from the plasma of PD patients into the striatum of mice and found that these exosomes were extensively taken up by microglial cells, activating them and promoting an inflammatory response. Building on these previous findings, Guo *et al.* 204 identified the mechanism by which microglia-derived exosomes promote the propagation and aggregation of α-syn in PD. Their findings suggest that targeting the release of microglia-derived exosomes or disrupting their propagation pathways could offer novel therapeutic targets for PD intervention. Additionally, previous studies have shown that exosomes secreted by astrocytes can induce protein aggregation in the mouse brain, suggesting that astrocytes may also play a role in the spread of misfolded proteins, such as α-syn 211. Wang *et al.* 212 discovered that pathological α-syn deposition impairs the lysosomal function of astrocytes, leading to the release of more α-syn-laden exosomes. These exosomes may then interact with neighboring neurons or glial cells, further propagating α-syn and promoting neurodegeneration. Thus, targeting the production, release, or cellular interactions of α-syn-carrying exosomes holds promise for developing more effective therapeutic strategies, offering new hope for patients with PD.

The pathophysiology of PD also involves oxidative stress, mitochondrial dysfunction, and neuroinflammation. α-syn can accumulate in mitochondria, generating high levels of ROS, which in turn induce the expression of inducible nitric oxide synthase (iNOS), leading to neuroinflammation and neuronal damage 163,213. Research has suggested that regulating oxidative stress may be promising therapeutic strategies for PD. One hypothesis is that intracerebral delivery of antioxidants could alleviate oxidative stress and protect neurons in PD patients. Catalase, a potent antioxidant, is unable to cross the BBB and is easily degraded 214.

However, studies by Haney *et al.* 136 demonstrated that exosomes loaded with catalase effectively degrade ROS and offer neuroprotection. Wang *et al.* 163 designed an engineered exosome capable of targeting damaged neurons and mitochondria in the PD microenvironment, characterized by high levels of ROS and iNOS. This exosome aims to achieve multistage therapy for PD by both inhibiting the progression of upstream factors (e.g., removal of α-syn aggregation) and repairing downstream damage (e.g., regulating ROS levels and repairing neuronal injury). Exosomes from serum also play a role in the pathogenesis of PD. For example, Jiang *et al.* 215 discovered that serum exosomes containing miR-137 help mitigate oxidative stress damage in PD neurons. Their experiments *in vitro* and *in vivo* showed that down-regulation of miR-137 upregulates oxidation resistance 1 (OXR1), which ameliorates oxidative stress-induced injury in PD. Additionally, exosomes derived from embryonic stem cells (T-MSC-Exos) can deliver miR-100-5p, which inhibits NADPH oxidase 4 (NOX4), reducing oxidative stress and ROS production via the Nox4-ROS-Nrf2 axis, thus improving dopaminergic neuronal damage in PD 216.

**Figure 6:**
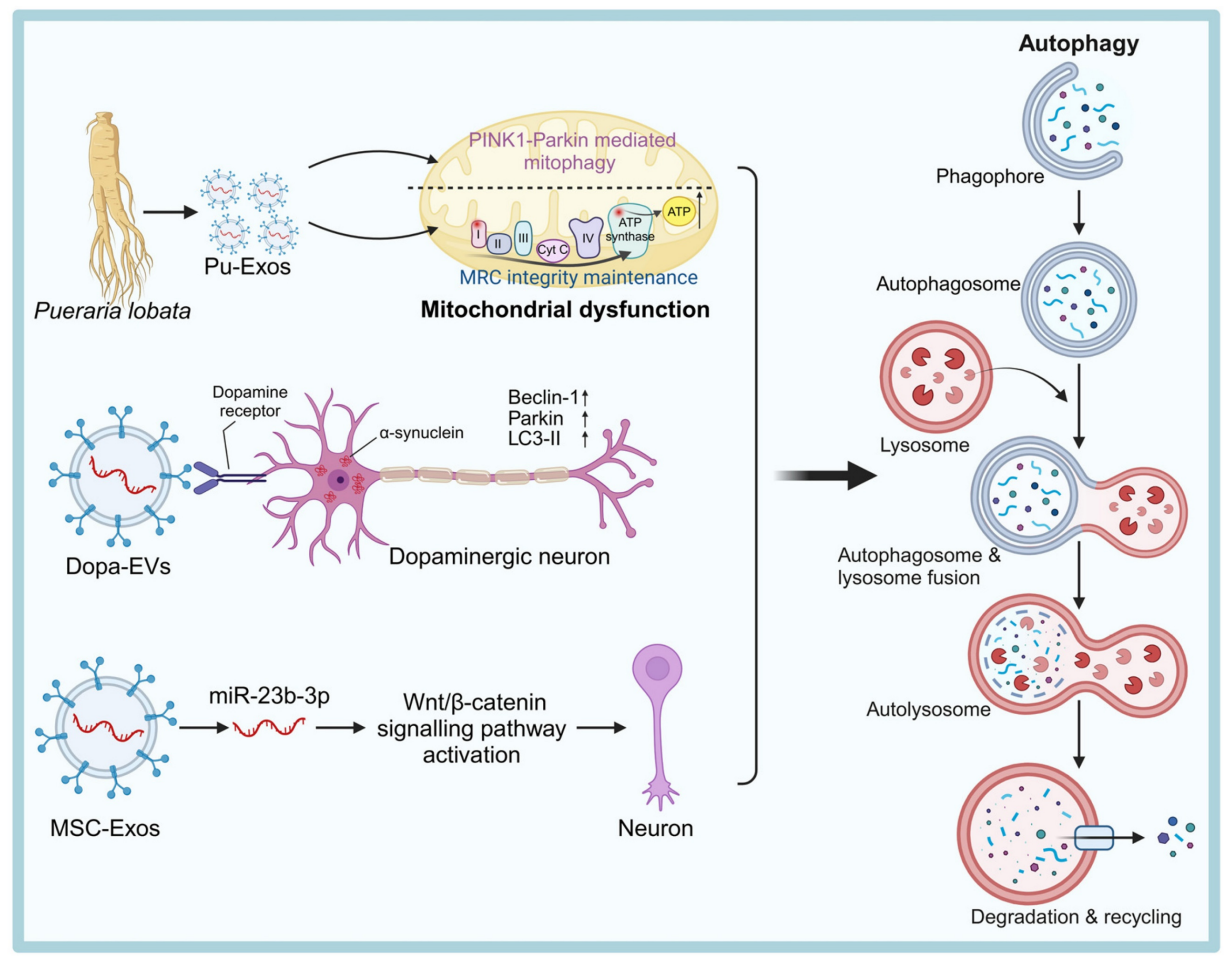
** Selected examples of exosomes as delivery vehicles for the treatment of PD via autophagy.** *Pueraria lobata* (Pu-Exos) restore dopaminergic neuron function by activating PINK1-Parkin-mediated mitophagy pathway; Dopa-EVs enhance clearance of misfolded proteins by upregulating key autophagy proteins including Beclin-1/Parkin/LC3-II; and MSC-Exos deliver miR-23b-3p to regulate Wnt/β-catenin signaling pathway, thereby inhibiting neurodegeneration. These exosome therapies collectively ameliorate PD pathology by enhancing cellular autophagy to eliminate dysfunctional mitochondria and misfolded proteins. The figure was created with BioRender. The original conceptual information is derived from published work, and were adapted with permission from 217, copyright 2024, Elsevier Ltd.

Exosomes can also be used to treat PD by inducing autophagy. By promoting autophagy, exosomes can help remove these toxic aggregates or dysfunctional mitochondria, thereby reducing cellular stress and increasing neuronal survival (Figure 6). Mitochondrial dysfunction is one of the pathological hallmarks of NDDs, and studies suggest that enhancing the mitophagy pathway may represent a novel therapeutic approach to target the potential pathogenic causes of NDDs 218.

For instance, Xu *et al.* 217 utilized exosomes derived from Pu-Exos to deliver therapeutic drugs to the brain, which modulated PINK1-Parkin-mediated autophagy and the activity of mitochondrial respiratory chain complexes. This approach reversed mitochondrial dysfunction in dopaminergic neurons, significantly alleviating both motor and non-motor symptoms in PD mouse models. In addition, Sul *et al.* 156 found that Dopa-EVs, aside from enhancing targeting efficiency, stimulate autophagy by upregulating the expression of Beclin-1, Parkin, and LC3-II, and promote the expression of tyrosine hydroxylase to alleviate dopaminergic neuron loss and motor dysfunction in a PD model. Geng *et al.* 219 demonstrated that MSC-Exos loaded with miR-23b-3p can modulate the Wnt/β-catenin signaling pathway, promoting autophagy in neurons and thereby mitigating the progression of PD.

In addition to modulating PD pathogenesis, researchers have also explored chemical delivery methods to improve dopamine transmission in the brain. One approach involves using exosomes loaded with dopamine or levodopa for intracerebral drug delivery. Although earlier attempts using nanocarriers to increase intracerebral drug delivery were not highly successful, Qu *et al.* 220 designed blood exosomes loaded with dopamine to target the brain. These exosomes cross the BBB via the transferrin-transferrin receptor interactions, resulting in more than a 15-fold increase in brain dopamine distribution. Animal studies have shown that dopamine-loaded blood exosomes improve dopaminergic neuron function, highlighting their potential for PD therapy.

#### Amyotrophic lateral sclerosis (ALS)

ALS is a fatal neurodegenerative disease, characterized by progressive skeletal muscle atrophy, myofascial fibrillation, and neurological impairments 5. The majority of patients die within 3-5 years of the first symptoms. The pathogenesis of ALS is not fully understood but involves impaired RNA metabolism, protein aggregation (such as DNA and RNA-binding protein and mutant superoxide dismutase 1 (SOD1)), autophagy defects, and mitochondrial dysfunction 221. Exosomes are being explored as therapeutic tools for ALS. For instance, Bonafede *et al.* 222 showed that exosomes derived from mouse adipose-derived stromal cells (ASC-Exos) protect motor neuron-like cells from oxidative stress by targeting mutant SOD1. Therefore, ASC-Exos could be a potential therapeutic approach for ALS.

Mitochondrial dysfunction, a key feature of ALS, can be alleviated by ASC-Exos 223. In an *in vitro* ALS model, ASC-Exos restored mitochondrial complex I activity, improved coupling efficiency, and membrane potential, which helped reduce mitochondrial dysfunction 224. Furthermore, ASC-Exos have been shown to improve motor performance and protect motor neurons in ALS mouse models, indicating their therapeutic potential.

#### Huntington's disease (HD)

HD is an autosomal dominant neurodegenerative disorder caused by the amplification of CAG repeats in the Huntington protein (HTT) gene 225. In normal individuals, the HTT gene contains 3-35 CAG repeats, while in individuals with HD, the HTT gene contains 36 or more CAG repeats 226. The resulting mutant HTT (mHTT) aggregates in neurons, leading to striatal cell death through transcriptional dysregulation, activation of the intrinsic apoptotic pathway, and mitochondrial dysfunction.

Zhang *et al.* Current therapeutic strategies for HD mainly focus on silencing mHTT through siRNAs and ASOs. Oligonucleotide therapeutics, which target DNA or RNA to block the expression of mutant proteins, are a promising approach in gene therapy 227. Recent studies have explored exosomes loaded with hydrophobically modified siRNAs (hsiRNAs) to specifically silence Htt mRNA 227. Zhang *et al.* 228 designed genetic circuits that modify the mouse liver to transcribe and self-assemble mHTT siRNA into RVG-modified exosomes. These exosomes significantly reduced mHTT expression and alleviated symptoms in HD mouse models.

Additionally, exosomes derived from ASCs have been shown to reduce mHTT aggregation in neurons 229. Studies have also indicated that mitochondrial dysfunction in HD is linked to the downregulation of the p-CREB-PGC1α pathway 230. ASC-Exos protect mitochondrial function by activating the p-CREB-PGC1α pathway, providing a potential therapeutic strategy for HD.

#### Multiple sclerosis (MS)

MS is an autoimmune disease affecting the CNS, primarily characterized by demyelinating lesions in the brain and spinal cord, axonal damage, and neuronal loss 231. Most treatment strategies for MS focus on preventing inflammation and promoting myelin regeneration.

Under normal physiological conditions, oligodendrocyte precursor cells (OPCs) differentiate into mature oligodendrocytes (OLs), which are responsible for forming myelin sheaths and ensuring the proper transmission of neural impulses 232. In MS, OPC differentiation is impaired, hindering the regeneration of myelin. Research has shown that MSC-Exos can enhance myelin regeneration by directly promoting OPC differentiation and indirectly modulating neuroinflammation in the CNS, thereby improving neurological function in MS models 149. BDNF plays a critical role in regulating the proliferation and differentiation of OPCs into OLs 233. Unfortunately, BDNF cannot cross the BBB and thus cannot be directly administered in MS treatments. Zhai *et al.* 234 developed RVG-modified exosomes (RVG-Exos) as a targeted drug delivery system to overcome this challenge. Their findings showed that RVG/BDNF-Exos successfully facilitated the delivery of BDNF to the brain, promoting OPC proliferation, OL differentiation, and myelin regeneration in the corpus callosum of cuprizone-induced mice, ultimately improving motor coordination. Additionally, triiodothyronine (T3), a bioactive molecule, has been shown to enhance myelination during CNS development. Exosomes loaded with T3, and modified with platelet-derived growth factor A (PDGFA) ligands, exhibit enhanced targeting ability for OPCs and OLs, thereby further promoting myelin formation 235.

BMSC-derived exosomes (BMSC-Exos) have also been shown to reduce demyelination, minimize CNS infiltration by inflammatory cells, and significantly improve neurobehavioral outcomes 236. After treatment with BMSC-Exos, levels of M2-associated cytokines (e.g., IL-10, TGF-β) were significantly increased, while levels of M1-associated cytokines (e.g., TNF-α, IL-12) were markedly reduced, further supporting the anti-inflammatory effects 237.

Building on previous IFN-based therapies for MS, researchers have speculated that exosomes derived from IFN-stimulated MSCs may offer enhanced therapeutic benefits. Riazifar *et al.* 57 assessed the efficacy of MSC-Exos and exosomes derived from IFNγ-stimulated MSCs (IFNγ-Exos) in MS treatment. Their results revealed that IFNγ-Exos significantly reduced demyelination and neuroinflammation while enhancing motor skills in experimental autoimmune encephalomyelitis mice. Moreover, IFNγ stimulation of dendritic cells (DCs) promoted the release of exosomes containing high levels of miR-219 238. These exosomes were effective in increasing myelin formation, reducing oxidative stress, and improving myelin regeneration following acute demyelination.

### Clinical Translation of Exosome Therapies: Trials and Challenges

**Figure 7:**
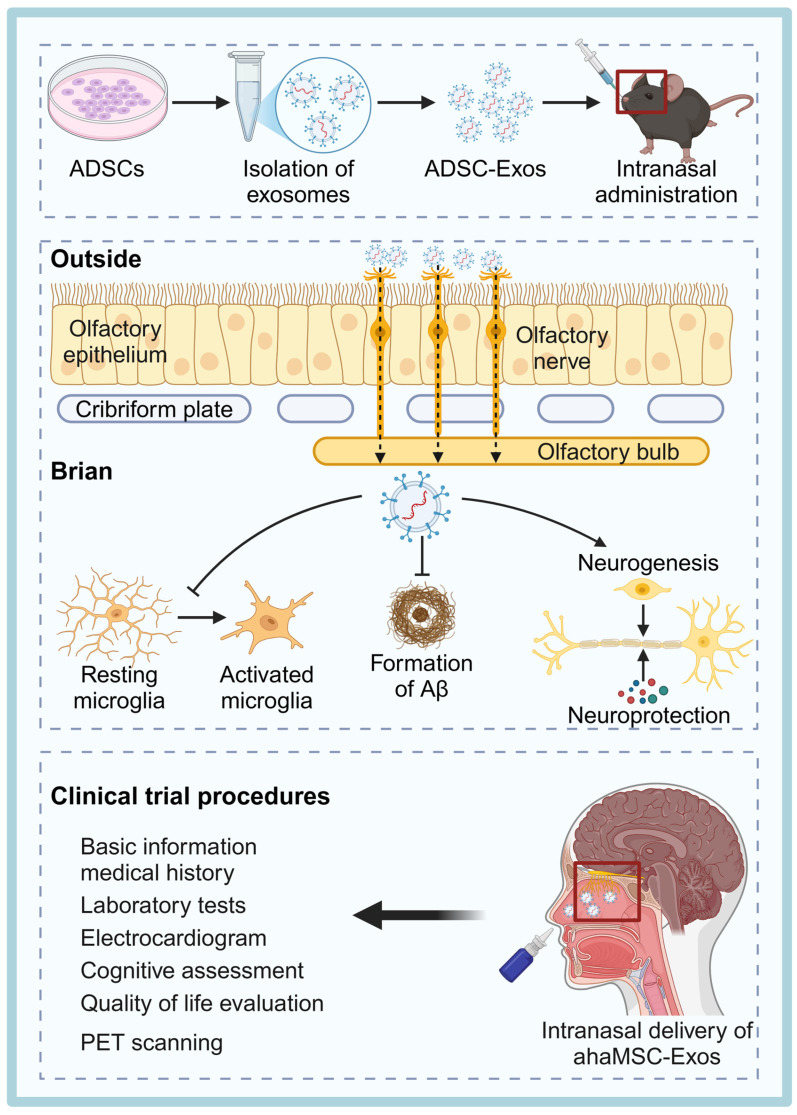
** Therapeutic mechanisms of ADSC-Exos in AD animal experiments and clinical trials of ahaMSC-Exos.** ADSC-Exos are delivered via the olfactory pathway (nasal mucosa→olfactory bulb) to target the brain, where they regulate microglial activation and Aβ production to exert neuroprotective effects. The figure also outlines the standardized clinical evaluation protocol (including cognitive tests and PET scans) for ahaMSC-Exos in clinical trials. This figure was created with BioRender.

Although basic research has demonstrated the significant therapeutic potential of exosomes in NDDs, clinical trials exploring exosomes as drug carriers remain relatively limited. A study by Ma *et al.* 239 showed that intranasal administration of ADSC-Exos enable rapid delivery to the brain via the olfactory nerve, alleviating memory deficits in APP/PS1 transgenic mice by reducing microglia activation, inhibiting Aβ deposition, and promoting neurogenesis and neuroprotection (Figure 7). Based on these findings, they conducted a Phase I/II clinical trial using allogeneic human ADSC-Exos (ahaMSC-Exos) in AD patients to assess its safety and efficacy 240. The results revealed improved cognitive function at 12 weeks, with a slight reduction in hippocampal volume in the medium-dose group, despite no significant changes in amyloid or Tau deposition. This study is the first international trial to use ahaMSC-derived exosomes for nasal administration in AD treatment, validating the safety of exosomal therapy and laying the foundation for future large-scale clinical trials. Akhlaghpasand *et al.* 241 conducted a first-in-human, single-arm, phase I clinical trial to evaluate the safety and potential effects of intrathecal injection of allogeneic exosomes derived from HUC-MSCs-Exos in patients with fully subacute SCI. The results of this phase I trial suggest that intrathecal injection of allogeneic HUC-MSCs-Exos is both safe and well-tolerated by patients. The findings indicate the need for a larger phase II clinical trial to further evaluate the feasibility and potential therapeutic benefits of this approach.

ADSC-Exos rapidly and efficiently enter the brain via intranasal administration, accumulating primarily in neurons of the central nervous system. Its therapeutic mechanism involves reducing microglia activation, inhibiting Aβ deposition, and promoting neurogenesis and neuroprotection, which together help alleviate the pathological manifestations of AD. In clinical trials, ahaMSC-Exos were used to assess their therapeutic efficacy in AD patients, with a focus on evaluating both safety and effectiveness.

Additionally, AB126 is an unmodified neurogenic exosome with the natural ability to cross the BBB and demonstrate anti-inflammatory and neuroprotective effects. In January this year, the U.S. FDA approved an Investigational New Drug (IND) application for AB126, making it the first exosome-based drug to enter human clinical trials. This approval marks a breakthrough in exosome drug delivery and provides new therapeutic hope for patients suffering from acute ischemic stroke.

Currently, most exosome therapies are still in Phase I clinical trials, focused on testing accelerated doses in a small cohort of patients to identify potential side effects. However, several clinical studies of exosome therapies for the treatment of NDDs have been initiated, with specific information from ClinicalTrials.gov (Table 6). Early findings suggest that exosomes are effective at transporting therapeutic molecules across the BBB, reducing neuroinflammation, and promoting nerve repair.

Although challenges such as large-scale production, standardization, and clinical efficacy validation remain, the potential of exosome therapies as precise and efficient treatments is promising. As research progresses, the clinical application of exosomes in treating neurodegenerative diseases is expected to accelerate, offering more effective treatment options for patients.

### The Importance of Interdisciplinary Collaboration: The Intersection of Nanotechnology, Neuroscience, and Clinical Medicine

In the rapidly advancing field of exosome-based therapies and other cutting-edge treatments for neurological and systemic diseases, interdisciplinary collaboration plays a pivotal role in fostering innovation and overcoming the complex challenges inherent in therapy development. Such collaboration has become a major trend for future advancements. For instance, Li *et al.* 242, addressing the current shortcomings in the treatment of neurological diseases, summarized key trends and provided in-depth insights into the innovation and development of neuromodulation technologies within the context of interdisciplinary cross-research. They identified three primary development trends and further explored the opportunities and challenges associated with applying and translating neuromodulation technologies in an interdisciplinary framework. In this context, we examine the importance of collaboration between three critical disciplines—nanotechnology, neuroscience, and clinical medicine—toward accelerating clinical trial progress and ensuring the successful translation of discoveries from the laboratory to clinical applications.

NDDs present a substantial economic and health burden worldwide. Despite ongoing research, there is still no effective strategy to cure these diseases. However, with the continuous advancement of nanotechnology, researchers have developed a variety of nanomaterials for brain tissue drug delivery, particularly the use of exosomes. Exosomes help overcome the limitations of conventional delivery systems, such as low bioavailability, instability, and rapid clearance, especially in sensitive areas like the brain. Nevertheless, focusing exclusively on nanotechnology development is insufficient to fully address the treatment of NDDs.

Neuroscience provides the foundational understanding of the molecular and cellular mechanisms underlying neurological disorders. It has also unveiled the critical mechanisms by which exosomes can be used therapeutically—such as penetrating the BBB and facilitating targeted delivery within complex neural networks. Only by gaining a deeper understanding of the brain's workings can the delivery systems of exosomes be optimized effectively. Finally, clinical medicine plays an essential role in translating these scientific findings into real-world treatments. Clinicians possess a practical understanding of patient needs, disease heterogeneity, and the challenges inherent in large-scale clinical trials. Their involvement ensures that new therapies are not only scientifically promising but also safe, effective, and feasible for a diverse range of patient populations.

In summary, while exosomes hold great potential as therapeutic delivery vehicles for treating NDDs, their successful application hinges on the interdisciplinary collaboration of nanotechnology, neuroscience, and clinical medicine. Through the deep integration and ongoing cooperation across these disciplines, researchers can share valuable resources—such as specialized laboratory equipment, patient data, and preclinical models—accelerating the process from basic discovery to clinical application. This continued collaboration will drive progress in emerging therapies like exosome-based treatments, ultimately opening new avenues for treating patients with neurological disorders and other diseases.

## Conclusion and Perspective

With an aging population, the incidence of NDDs is rising, making early intervention crucial. Recent progress in nanomedicine has deepened our understanding of exosomes, which are a promising class of EVs for drug delivery due to their excellent biocompatibility, low immunogenicity, ability to cross the BBB, prolonged circulation time, and diverse cargo-loading capacity. They can not only efficiently transport biomolecules (e.g., proteins, nucleic acids) to brain targets, but also enhance inter-neuronal communication. Notably, ISEV updated the definition of EV in the MISEV2023 guideline by deleting the expression “naturally released” 120, to avoid inadvertently excluding engineered EVs or EVs generated under different cell culture conditions, and to expand the theoretical framework for their therapeutic applications. Based on these properties, exosomes are considered as a potential game-changer for treating AD, PD, ALS, HD and MS, and several clinical trials are underway to evaluate their safety and efficacy.

However, the clinical translation of exosome therapy still faces multiple challenges. Current exosome isolation techniques can hardly meet the high requirements for yield, purity and batch consistency in clinical applications. Surface modification of exosomes, while enhancing BBB penetration and intracerebral drug delivery, may produce off-target effects and reduce reproducibility. In addition, existing cargo loading techniques, such as incubation, electroporation and sonication, still face limitations such as cost, efficiency and potential damage to exosomes. To overcome these challenges, the development and optimization of novel technologies is critical to advance exosome-based drug delivery systems. In addition, we need to deepen our understanding of exosomes' biogenesis, release mechanisms, and trans-BBB transport processes, which are still only partially understood, limiting their full therapeutic potential. Clinical trials aimed at validating the safety and efficacy of exosome therapies also need to be improved in terms of dosage selection, control group settings, efficacy assessment and safety monitoring. Standardized assessment metrics are essential for accurately determining treatment outcomes and ensuring patient safety (Table 7).

Currently, exosomes are regulated as biological products in many countries and regions. However, due to the complexity of exosomes, the regulatory environment worldwide exhibits complexity and variability. The regulatory standards in different regions are mainly based on the differences in the characteristics of exosomes: in the United States and Europe, the regulatory focus is on whether the content of exosomes affects the physiological functions of the human body; while in Japan, South Korea, and Taiwan, China, the regulatory rules are mainly based on the way of obtaining exosomes 243. Moreover, there are also differences in the implementation of GMP quality systems in various countries, leading to differences in regulatory approaches. Current GMP (cGMP) focuses more on quality control of the final product, whereas the Pharmaceutical Inspection Agreement and Co-operation Scheme GMP (PIC/S GMP) emphasizes more detailed organization, validation, and training requirements, and differs especially in the quality assessment of cellular products. This difference in regulatory focus is a direct result of the lack of harmonization of standards for exosome manufacturing. Developers face a lack of clear guidance on product classification, evaluation criteria, and approval pathways, contributing to uncertainties in development timelines. Without a coordinated international regulatory framework, exosome therapies—despite their promising potential—may remain trapped in the bottleneck between laboratory research and clinical translation.

Beyond technical and regulatory issues, other factors such as cost, regulation, and large-scale production must be addressed for the successful clinical application of exosome therapies. Interdisciplinary collaboration between materials science and neuroscience is pivotal in overcoming these obstacles. Expertise from materials science can lead to the design of advanced materials that improve exosome stability, targeting efficiency, and cargo-loading capacity, while neuroscience can offer insights into the mechanisms governing exosome interactions with the brain. By combining knowledge from these two fields, we can develop more effective exosome therapies, reduce production costs, and meet regulatory standards, ultimately enabling widespread clinical use for treating NDDs.

Looking ahead, several areas hold promise for future technological breakthroughs in exosome-based therapies. Advances in nanomaterial design can help optimize exosome isolation and purification processes, enhancing scalability while maintaining high quality. Surface engineering could improve targeted delivery and reduce off-target effects, while cargo-loading technologies—such as synthetic biology or advanced electroporation—may increase exosome cargo capacity without compromising integrity. Furthermore, the integration of gene editing techniques could allow for precise control over exosome content, opening the door to tailored therapeutic applications.

In conclusion, short-term efforts should focus on ensuring the safety of exosome therapies through comprehensive Phase I, II, and III clinical trials to assess their tolerability and preliminary efficacy in treating NDDs. Long-term objectives should include refining production processes, enhancing cargo-loading capacities, and gaining a deeper understanding of exosome biogenesis and BBB crossing mechanisms. With sustained interdisciplinary collaboration and technological advancements in both materials science and neuroscience, exosomes hold the potential to revolutionize the treatment of NDDs.

## Figures and Tables

**Table 1:** Comparison of the performance of different nanomaterials in drug delivery

	Nanomaterials	Exosomes	Liposomes	Metal nanoparticles	Polymeric nanoparticles	Ref.
Characterization						
Biocompatibility		High biocompatibility, excellent biotolerance	Generally good, dependent on lipid composition	Lower, potential toxicity from metal ions	High, can be improved by modifying the polymer	16
Targeting ability		Excellent targeting ability, which can be further enhanced through surface modification technologies, allowing for precise drug delivery	Moderate, can be improved through surface modifications	Moderate, requires surface modifications to enhance targeting	Good, targeting capability can be improved through surface functionalization	17
Stability		Moderate stability, susceptible to environmental factors (e.g., pH, temperature); however, some exosomes are optimized to withstand changes in these conditions effectively	Easily affected by environmental factors (e.g., temperature, pH), especially during prolonged storage or transport, leading to rupture or leakage and reduced drug efficacy	Good, but may have issues with dissolution or oxidation	High stability, adjustable as needed, but may degrade or lose function during prolonged storage, making it unsuitable for complex drug delivery systems in brain tissue	18-20
Drug loading capacity		High, can load a variety of drugs (proteins, RNA, etc.)	Moderate, suitable for encapsulating both water-soluble and lipophilic drugs	High, capable of loading various drug molecules	Relatively high, suitable for loading small molecules and biomacromolecules	16
Drug release control		Adjustable, biodegradable, can achieve sustained release, can also be combined with hydrogels for controlled release	Controllable, external stimuli (e.g., pH, temperature) can be used to control release	Limited, typically not suitable for controlled release	Adjustable, especially suitable for long-term release systems	18
Immunogenicity		Low immunogenicity, enabling better immune system evasion and ensuring the safety of long-term use	Lipid bilayer helps immune evasion	Metal nanoparticles are highly immunogenic and may provoke an excessive immune response. Their accumulation in the body can lead to organ damage, which makes clinical translation challenging	Potential for immune response, especially when polymer materials are large	19
Production and purification difficulty		High, extraction and purification are complex	Low, production technology is mature but depends on lipid stability	Low, production is simple, but purity needs to be monitored	Moderate, synthesis and purification technologies are constantly evolving	21
Membrane penetration ability		Excellent, able to cross the BBB, cell membranes, etc	Moderate, can be enhanced by modifications	The BBB is poorly penetrated, limiting its effectiveness in the treatment of NDDs	Moderate, can be improved by special modifications, but this challenge is often difficult to overcome in clinical translation	22
Application advantages and scope		Suitable for the delivery of RNA, proteins and other biomolecules with natural targeting ability, which can be widely used in a variety of NDDs	Suitable for small molecule drugs, widely used in vaccine delivery, gene therapy	Suitable for metal ion transport and special treatments, currently used in antimicrobial, cancer therapy, imaging applications	Can design different carrier systems for a variety of drugs, mainly for tumor therapy and gene therapy applications	23

**Table 2:** Sources of exosomes

Exosome source	Advantages	Disadvantages	Main fields of application	Ref.
Mesenchymal stem cells (MSCs)	Highly biocompatible; low immunogenicity; carries a variety of neuroprotective factors that can reduce neuroinflammation and promote neurogenesis; surface modifications can further improve targeting and drug-carrying capacity	Higher production costs, more challenging to control exosome purity	Used in tissue repair and regeneration, treatment of neurodegenerative diseases (e.g., AD, PD, ALS), spinal cord injury, immune modulation, cardiovascular diseases, and lung disorders, and have been extensively applied in clinical trials for NDDs	45-47
Neural stem cells (NSCs)/neurons	Naturally target the nervous system, high biological barrier penetration, low immunogenicity	Difficult to produce on a large scale, limited source of cells, long purification time, unclear dosage and mechanism of action, therefore less used in clinical trials	Mostly used for NDDs, strokes, spinal cord injuries, etc	48,49
Induced pluripotent stem cells (iPSCs)	These exosomes are capable of differentiating into various neural cell types and are rich in factors that promote cell repair and neuroprotection. With strong neurorepair potential and low immunogenicity, they can be tailored to the patient's genetic background, providing more personalized treatment options and showing considerable clinical translation potential	Technically complex, costly and potentially ethically controversial	Can be used for cardiovascular disease, nervous system, limb disease, liver disease, skin disease, etc	50,51
Endothelial cells (ECs)	Easy to obtain and culture, activates blood vessel formation	There is a risk of immune response, potential insufficient targeting of the nervous system, and the need for precise isolation and purification, which increases production complexity and costs. Additionally, their limited ability to effectively cross the BBB restricts their potential for brain-targeted therapies, making clinical translation challenging	Used in tissue regeneration, angiogenesis, cellular regeneration, and skin repair, with significant potential in the fields of regenerative medicine and tissue healing	52,53

**Table 3:** Methods of exosome isolation

Technique	Principle	Advantages	Disadvantages	Application scenarios	Ref.
Differential ultracentrifugation	Sedimentation coefficients for exosomes and other substances	Easy to operate, high yield, high separation purity	Time consuming, non-specific, poor reproducibility, risk of exosome rupture	High yield, suitable for exosome function studies	68,69
Density gradient ultracentrifugation	Densities	High purity	The steps are cumbersome	Typically used to study and isolate specific subpopulations of exosomes	70,71
Ultrafiltration	Volume and molecular weight	Simple and fast operation	Many steps, time-consuming, membrane holes prone to clogging	Suitable for rapid isolation of exosomes from complex mixtures when high throughput is required	72
Size exclusion chromatography	Pore size of gels	Simple operation, good reproducibility, large sample size	Low purity, requires further purification	Suitable for applications where reproducibility and large sample sizes are required but purity is secondary	73,74
Polymer-based precipitation separation	Hydrophobicity	Small sample size required, easy to operate, no need for expensive or specialized equipment	Low specificity, contaminated with free proteins	Used for small-scale separations, they are simple and inexpensive, and are best suited for screening or where specific purity is not required	75
Immunoaffinity separation	Affinity	High purity, specificity and low time consumption	Reagents are expensive and cannot be applied on a large scale	Suitable for applications requiring high purity and specificity, ideal for clinical or targeted research environments	76,77
Microfluidics-based technology	Immunoaffinity, acoustic wave, dielectrophoresis, size, magnetism	Fast, high-throughput, and low sample/reagent consumption	Requires electric heating, low resolution, low purity	Suitable for high-throughput applications with small sample sizes, best suited for lab-scale research and diagnostics	78-80

**Table 4:** Exosome characterization parameters

Characterization parameter	Criteria	Method	Roles in the GMP process
Physical characteristic	Particle number, size, morphology, zeta potential	Nanoparticle tracking analysis (NTA), NanoFCM, Super resolution microscopy, Tunable resistive pulse sensing (TRPS), Dynamic light scattering (DLS), Flow cytometry, Scanning electron microscopy (SEM), Transmission electron microscopy (TEM)	Ensure the quality consistency of exosomes. Particle size directly affects the distribution, targeting and biological activity of exosomes *in vivo*; zeta potential provides information on the surface charge and stability of exosomes; morphological observation of whether there is a typical teacup holder structure and the integrity of the membrane structure ensures its normal functioning
Biochemical composition	Total Protein, lipids, nucleic acids, surface markers, non-protein markers	Western Blot, Mass Spectrometry, Colorimetry, Proteomic analysis, ELISA, Fourier transform infrared spectroscopy (FTIR), Reverse transcription real-time quantitative PCR (RT-qPCR), BCA	Accurate quantification of biomolecules in exosomes guarantees both the stability of bioactive components and exosome specificity, ensuring batch-to-batch quality consistency. This approach also enables drug-loading capacity assessment and targeting capability studies
Purity and integrity	Particle number/total protein, total protein/total lipid, particle number/total lipid, particle number/total nucleic acid, total protein/total nucleic acid	NTA, Nano-Flow Cytometry (NanoFCM), Triton X-100 lysis: ratio of particles before and after membrane-breaking treatment, Measurement of membrane dye, BCA, TEM	Ensure activity and function, reduce immunogenicity, and precisely control the dosage administered in clinical trials to ensure the accuracy and reproducibility of results
Security	Viruses, bacteria, fungi, mycoplasma, endotoxins of internal and external origin	qPCR, Gel Clot Method of Limulus Amebocyte Lysate, Culture method ELISA, Next Generation Sequencing (NGS)	Ensure exosome product quality, prevent contamination, ensure purity and clinical application safety
Cargo stability	miRNA integrity, protein activity, RNA fragmentation	Bioanalyzer, qPCR, enzymatic activity assays	Ensure therapeutic efficacy, maintain batch consistency, and ensure safety of exosomal products
Functional validation	Cellular uptake, bioactivity, BBB penetration	Cell uptake assay, fluorescence microscopy, *in vitro* BBB models, *in vivo* biodistribution	Ensure product quality and stability, guide clinical dosing, assess drug delivery efficiency, and predict clinical efficacy and safety

**Table 5:** Exosomal drug loading technologies

Technique	Procedure	Tested Cargo	Advantages	Disadvantages	Potential for clinical application	Ref.
Electroporation	Forming restorable micropores on the exosome surface through the action of an electric field	siRNAs, microRNA, DNA, mRNA, proteins	Easy to operate; less influence on the receptor-ligand on the exosome surface; suitable for macromolecules	Induces aggregation of exosomes or contents and reduces drug loading efficiency	Medium: Already used in some preclinical studies, requires a solution by adjusting the electric field strength, the perforation time or using special surface modification techniques to improve the drug-carrying efficiency	129,130
Sonication	Sonication of homogenization probes for exosomes and drug mixtures	Hydrophilic drug molecules, proteins	High drug loading efficiency; Sustainable drug release	Exosomes tend to aggregate and affect their surface protein structure	Medium: suitable for high drug loads	131
Freeze-thaw	Rapid freeze -thaw cycles at < -80°C	siRNAs, microRNA, proteins and macromolecules	Simple operation; Not easy to destroy bioactive substances	Exosomes are easily aggregated and have low encapsulation efficiency	Low: temperature and freeze-thaw cycles can be adjusted to reduce aggregation and improve encapsulation efficiency	132
Saponification	Saponins and other chemicals create pores in exosomes	Hydrophobic drugs, DNA/RNA and proteins, catalase	High drug loading efficiency	Affects exosome stability; Chemical reagents are difficult to remove; Risk of toxicity	Low: toxicity risks and stability issues hamper their clinical translation	133
Extrusion	Liposome extruder extrusion for exosomes and drug mixtures	miRNA, siRNAs, curcumin, catalase	High efficiency: Homogeneous particle size of exosomes obtained	Mechanical forces may alter the nature of the exosome membrane (e.g., membrane protein structure) and loss of contents	High: already used in several clinical trials, but there is a risk of membrane damage and further optimization of extrusion equipment is required	134
Co-incubation	Exosomes/parental cells and drug co-incubation at room temperature	Minor molecule chemicals, proteins, siRNAs, curcumin, microRNA	Easy to operate	Low loading efficiency	Medium: simple and effective for small molecules, but requires improved incubation conditions such as temperature, drug concentration and incubation time to increase drug loading efficiency	135-137

**Table 6:** Clinical trials related to exosomes

Disease	Trial phase and sample size	Primary objective	Experimental method	Preliminary results	Advantages of this exosome	ID
AD	Interventional I/II 9	To evaluate the safety and efficacy of allogeneic human adipose-derived MSC exosomes (ahaMSCs-Exos) in patients with mild to moderate AD	Nasal administration of different doses of ahaMSCs-Exos.	Nasal administration of ahaMSCs-Exos is safe and well-tolerated, with doses of at least 4×10^8 particles considered suitable for further clinical trials	ahaMSCs-Exos exosomes have better immunomodulatory and neuroprosthetic effects, adipose tissue is easily available and less likely to cause immune rejection	NCT04388982
ALS	Interventional I 38	To assess the safety and preliminary efficacy of nasal exosomes derived from human umbilical cord MSCs (hUC-MSC-sEV-001) in ALS	Nasal administration	Ongoing	hUC-MSC-sEV possesses immune-modulatory and neuroprotective effects, and umbilical cord-derived MSCs are easier to obtain and have lower immunogenicity compared to adult-derived MSCs	NCT06598202
NDDs	Interventional I 100	To assess the safety and preliminary efficacy of nasal hUC-MSC-sEV-001 exosomes derived from human umbilical MSCs in various neurodegenerative diseases	hUC-MSC-sEV-001 intranasal administration	Ongoing	Strong neuroprotective ability to cross the blood-brain barrier and repair neurological damage	NCT06607900
NDDs	Interventional Not Applicable 300	To evaluate the safety and efficacy of exosome delivery combined with synchronized transcranial focused ultrasound for treatment-resistant depression, anxiety, and neurodegenerative dementia	Focused ultrasound delivery of intravenously infused exosomes	/	Can effectively enhance drug delivery in the brain and break the blood-brain barrier	NCT04202770
PD	Observational 601	To identify exosomal protein biomarkers related to PD susceptibility and/or progression in PD patients, and to establish targeted effect detection methods for future LRRK2 inhibitor clinical trials	/	/	The choice of exosome biomarkers as an early diagnostic tool for PD enables non-invasive detection of pathological features in PD patients, facilitating early intervention and monitoring of efficacy	NCT01860118
PD	Observational 38	To optimize the protocol for isolating exosomes from CSF to improve the detection of LRRK2 activity in human CSF	Lumbar puncture for CSF collection	/	CSF- Exos directly reflect CNS status and are suitable for clinical monitoring of PD	NCT03775447
Refractoryfocal epilepsy	Interventional Early I 34	To assess the safety, tolerability, and preliminary efficacy of nasal exosomes (GD-iEXo-002) derived from iPSC in treating refractory focal epilepsy	iPSC-derived exosomes administered nasally	Ongoing	iPSC-Exos have strong neuroprosthetic and protective properties, can promote nerve regeneration, and can be used in the clinic without rejection	NCT05886205
Acute ischemic stroke	Interventional I 29	To evaluate the safety and preliminary efficacy of intravenous exosomes derived from human iPSC (GD-iExo-003) in acute ischemic stroke	Intravenous administration of GD-iExo-003 and placebo	Ongoing	iPSC-Exos have neuroregenerative capacity to repair brain tissue damage after acute ischaemic stroke and are more immunotolerant compared to adult-derived exosomes	NCT06138210

**Table 7:** Standardized assessment indicators for exosome treatment in NDDs

Indicator Category	Standardized Indicators	Ref.
Cognitive function	Alzheimer's Disease Assessment Scale-Cognitive Subscale (ADAS-Cog) Mini-Mental State Examination (MMSE) Montreal Cognitive Assessment (MoCA) Neuropsychiatric Inventory (NPI)	244-247
Motor function	Movement Disorders Society Unified Parkinson Disease Rating Scale (MDS-UPDRS) Timed Up and Go Test (TUG)	248
Neuroimaging	Positron emission tomography (PET) Magnetic Resonance Imaging (MRI) Functional MRI (fMRI)	249
Quality of life	Alzheimer's Disease Cooperative Study-Activities of Daily Living (ADCS-ADL) Instrumental Activities of Daily Living Scale (IADL) Parkinson's Disease Questionnaire (PDQ-39)	250,251
Inflammation level	TNF-α, IL-1, IL-6, Aβ, Tau	240
